# Sleep Deprivation Activates a Conserved Lactate‐H3K18la‐RORα Axis Driving Neutrophilic Inflammation Across Species

**DOI:** 10.1002/advs.202504028

**Published:** 2025-07-21

**Authors:** Ren Zhou, Keyun Li, Xiezong Hu, Shuhao Fan, Yuxuan Gao, Xiaoshu Xue, Yu Bu, Haoyi Zhang, Yili Wang, Chunjiao Wei, Shangrong Zhang, Zhongwen Xie, Chao Liu, Peng Chen, Zongjun Yin, Dalong Ren

**Affiliations:** ^1^ College of Animal Science and Technology Anhui Agricultural University Hefei 230036 China; ^2^ Anhui Province Key Laboratory of Embryo Development and Reproductive Regulation Fuyang Normal University Fuyang 236037 China; ^3^ School of Life Sciences Suzhou Medical College of Soochow University Suzhou 215123 China; ^4^ Institute of Brain Science The First Affiliated Hospital of Anhui Medical University Hefei 230022 China

**Keywords:** inflammation, lactylation, neutrophil, Rorα, sleep deprivation

## Abstract

Sleep deprivation critically disrupts physiological homeostasis, impairing development, metabolic balance, and immune regulation, with excessive neutrophil activation being a hallmark consequence. However, the molecular mechanisms underlying sleep deprivation‐induced neutrophilic inflammation remain elusive. Here, it is shown that acute sleep deprivation in mice triggers neutrophil hyperactivation, resulting in aberrant peripheral accumulation and a systemic cytokine storm. Mechanistically, this pathology is driven by metabolic dysregulation, specifically, increased glycolytic flux, which elevates tissue lactate levels and enhances histone H3K18 lactylation. Through H3K18 lactylation‐specific CUT&Tag profiling, pronounced lactylation enrichment is identified at the promoter of the *Rorα* gene, directly activating its transcription. Genetic ablation of *Rorα* or pharmacological inhibition of glycolysis attenuate neutrophil recruitment and mitigated inflammation in sleep‐deprived zebrafish. Strikingly, this metabolic‒epigenetic axis is evolutionarily conserved, as demonstrated by the recapitulation of key findings in diurnal zebrafish and pigs. The study reveals a lactate‐H3K18 lactylation‐Rorα signaling cascade that links sleep deprivation to immune dysregulation, suggesting actionable targets for combating sleep‐related inflammatory disorders.

## Graphical abstract

Sleep deprivation triggers systemic immune activation characterized by neutrophil accumulation and cytokine release. This study reveals a conserved metabolic‒epigenetic mechanism whereby lactate‐induced H3K18 lactylation upregulates RORα expression, promoting neutrophilic inflammation. Findings across mice, zebrafish, and pigs highlight lactylation as a key mediator of sleep loss‐induced immune dysregulation.

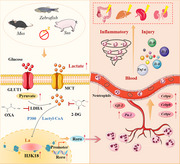

## 1 Introduction

Sleep serves as a cornerstone of physiological homeostasis, critically supporting immune balance, cognitive function, and metabolicregulation.^[^ ^1^, ^2^ ^]^ In modern societies, chronic stress and environmental disruptions have normalized sleep deprivation, a condition increasingly implicated in immune dysfunction, metabolic syndrome, and chronic inflammatory disorders.^[^ ^3^ ^]^ Mounting evidence highlights its systemic consequences, including circadian rhythm disruption, impaired immune surveillance, and aggravated pathologies such as obstructive sleep apnea.^[^ ^4^, ^5^, ^6^, ^7^ ^]^ Additionally, sleep deprivation impedes the clearance of neurotoxic metabolites (e.g., amyloid‐β)^[^ ^8^, ^9^ ^]^ and promotes oxidative stress as well as excessive production of proinflammatory cytokines, such as interleukin‐1β (IL‐1β) and tumor necrosis factor‐α (TNF‐α).^[^ ^10^, ^11^, ^12^, ^13^ ^]^ SD has been shown to disrupt immune homeostasis by promoting systemic inflammation, increasing the number of circulating neutrophils, and increasing the levels of proinflammatory cytokines such as IL‐6 and IL‐17A. Recent studies have also suggested that brain‐derived PGD2 efflux and activation of the peripheral PGD2/DP1 axis contribute to immune activation following SD.^[^ ^14^ ^]^ Despite these associations, the molecular mechanisms underlying sleep deprivation‐induced immune dysregulation, particularly through innate immune cell dysfunction, remain poorly understood.

Neutrophils, as frontline innate immune cells, orchestrate rapid antimicrobial responses through reactive oxygen species (ROS) generation, degranulation, and neutrophil extracellular trap (NET) formation.^[^ ^15^, ^16^, ^17^, ^18^ ^]^ Recent studies have revealed that neutrophils dynamically reprogram their metabolism to meet the bioenergetic demands of immune activation, with glycolysis emerging as a central driver of their effector functions.^[^ ^19^ ^]^ McAlpine et al. reported that sleep fragmentation suppresses hypothalamic hypocretin, elevates bone marrow CSF1 and Ly6C^high^ monopoiesis, and thereby accelerates atherosclerosis—establishing a neuroimmune axis that connects disturbed sleep to hematopoietic dysregulation and cardiovascular disease.^[^ ^6^ ^]^ Notably, lactate, a glycolysis byproduct, has evolved from a metabolic waste product to a signaling molecule that regulates gene expression via histone lactylation, a novel posttranslational modification.^[^ ^20^ ^]^ Specifically, histone H3K18 lactylation has been shown to activate immune‐related gene transcription, suggesting an epigenetic mechanism that may modulate neutrophil activity.^[^ ^21^ ^]^ However, whether sleep deprivation influences neutrophil metabolism or histone lactylation‐mediated epigenetic regulation remains unexplored.

Critically, sleep deprivation disrupts systemic metabolic homeostasis through multiple pathways, including dysregulation of metabolic hormones (e.g., leptin and ghrelin),^[^ ^22^, ^23^ ^]^ gut barrier impairment, and microbiota alterations,^[^ ^24^, ^25^, ^26^, ^27^, ^28^ ^]^ all of which synergize to fuel systemic inflammation. These perturbations likely extend to neutrophils, altering their metabolic states. For example, elevated glycolytic flux during sleep deprivation may increase lactate accumulation, potentially driving histone lactylation and subsequent activation of inflammatory pathways. Nevertheless, the causal relationships among sleep deprivation, neutrophil metabolic reprogramming, epigenetic modifications (e.g., H3K18 lactylation), and dysregulated immune responses remain unclear.

We hypothesize that sleep deprivation induces a shift in neutrophil metabolism toward enhanced glycolysis and lactate accumulation, which promotes histone H3K18 lactylation to activate Rorα‐dependent transcriptional programs, thereby exacerbating neutrophilic inflammation. By integrating transcriptomic, epigenomic, and functional analyses, this study delineates a metabolic‒epigenetic axis linking sleep deprivation to immune dysregulation. Our findings not only provide mechanistic insights into sleep‐related pathologies but also identify potential therapeutic targets for inflammatory diseases associated with sleep deprivation.

## 2 Results

### 2.1 Establishment of Sleep Deprivation Models in Mice, Zebrafish, and Pigs

**Figure 1:**
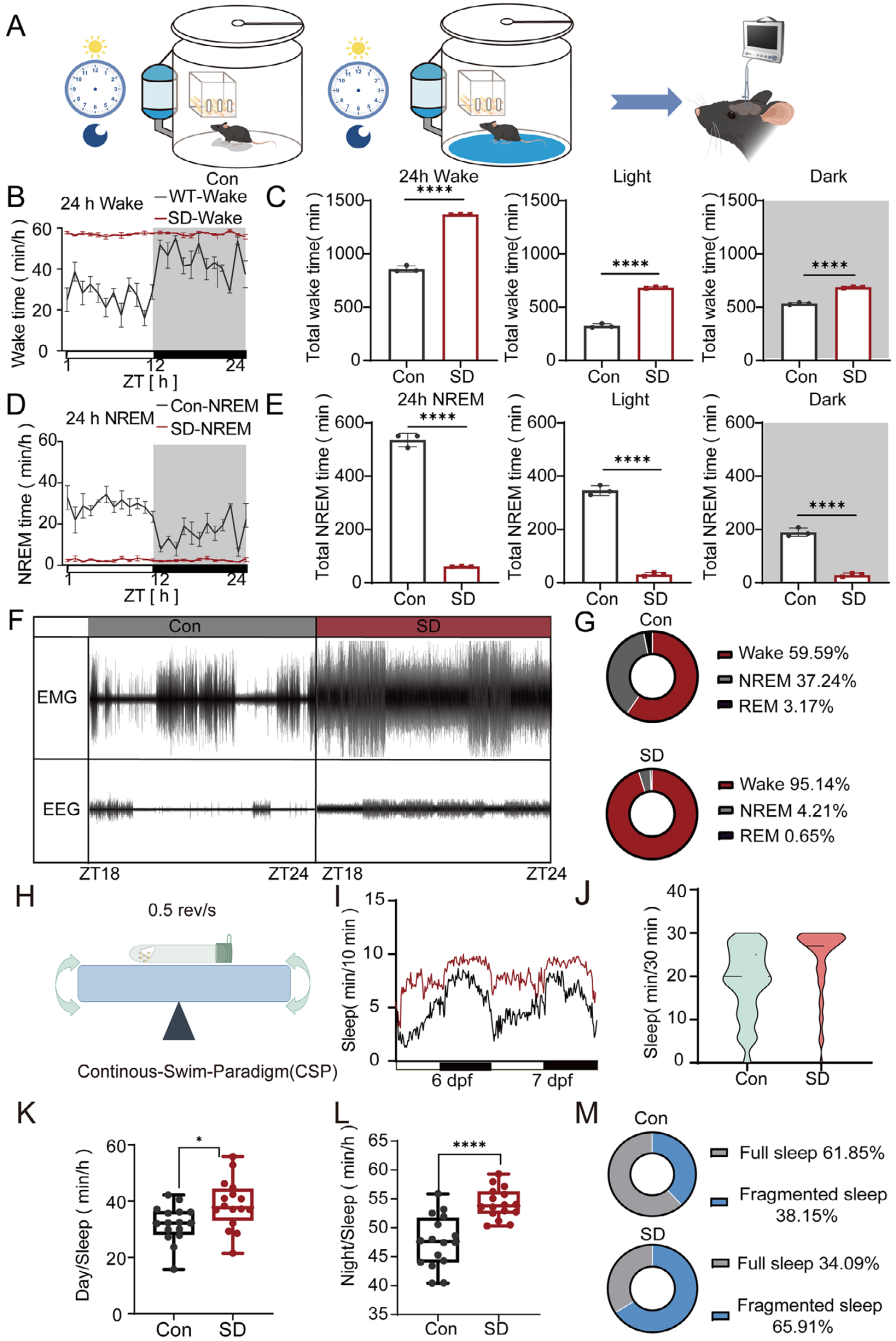
Sleep deprivation models in mice and zebrafish. A) Schematic diagram of the sleep deprivation paradigm in mice. The mice were either maintained under normal conditions or subjected to 24‐h sleep deprivation via the CPW method. B) Distribution of wake time across a 24‐h period in control and sleep‐deprived mice (*n* = 3). The data represent the time spent in the wake state throughout the light/dark cycle. C) Total wake time in 24 h, separated by light and dark phases, showing the impact of sleep deprivation on wakefulness in mice. D) Distribution of NREM sleep time across a 24‐h period for Con and SD mice, illustrating changes in sleep patterns due to SD. E) Total NREM sleep duration in the 24‐h, light, and dark phases, demonstrating a reduction in NREM sleep following sleep deprivation. F) Representative EEG and EMG traces of both Con and SD mice. The traces illustrate the typical patterns of brain and muscle activity during sleep. G) Percentages of time spent in the awake, NREM, and REM states in Con and SD mice over a 24‐h period. The data reveal the shift in sleep architecture due to SD. H) Schematic diagram of the CSP used to induce sleep deprivation in zebrafish. Zebrafish were subjected to continuous swimming conditions to simulate sleep disruption. I) Sleep distribution over 24 h following sleep deprivation in zebrafish (*n* = 24). The plot shows a distinct alteration in sleep patterns compared with those of the control group, indicating the effects of prior 24‐h SD exposure. J) Violin plot comparing sleep duration within a 30‐min interval between Con and SD zebrafish, highlighting the reduction in sleep efficiency after SD. K) Total day sleep duration in Con and SD zebrafish, showing the effect of SD on daytime sleep in zebrafish. L) Total night sleep duration in Con and SD zebrafish. M) The percentage of full sleep and fragmented sleep episodes in Con and SD zebrafish, emphasizing the increase in fragmented sleep in SD zebrafish. Statistical significance was analyzed at each time point via t tests, with significance levels denoted as follows: ns, *p* > 0.05; *, *p* < 0.05; **, *p* < 0.01; ***, *p* < 0.001; ****, *p* < 0.0001.

To investigate the impact of sleep deprivation (SD) on neutrophilic inflammation, we established SD models in mice, zebrafish, and pigs. In mice, SD is induced via the curling prevention by water (CPW) model,^[^ ^14^ ^]^ with sleep–wake states monitored via electroencephalography (EEG) and electromyography (EMG) (**Figure** **1**A). Sleep deprivation (SD) significantly increased total wakefulness in the mice, with the animals remaining awake for an average of ≈1370 min per day, accounting for 95.14% of the observation period, compared with ≈900 min (59.59%) in the control group (Figure 1B,C). SD also markedly altered sleep architecture by reducing both nonrapid eye movement (NREM) and rapid eye movement (REM) sleep during both the light and dark phases (Figure 1D,E). EEG and EMG recordings revealed that sleep patterns in SD mice were disrupted, characterized by fragmented NREM sleep and attenuated REM sleep signals (Figure 1F). In addition, compared with control mice, SD mice presented a significant increase in wakefulness (95.14%) and drastic reductions in NREM sleep (from 37.24% to 4.21%) and REM sleep (from 3.17% to 0.65%) (Figure 1G). These findings indicate that SDs induce prolonged wakefulness, sleep fragmentation, and significant disturbances in sleep homeostasis.

In zebrafish, SD was induced via a continuous swimming protocol (CSP) as previously described by Leung et al.^[^ ^29^ ^]^ Sleep patterns were assessed at 5 days postfertilization (dpf) following 24 h of deprivation. Owing to technical constraints, behavioral assessments were conducted during the postdeprivation recovery phase. Consistent with the findings of Louis et al., sleep rebound was observed in zebrafish after SD. Notably, sleep in zebrafish has been well characterized via established behavioral criteria: both adult and larval zebrafish exhibit circadian‐regulated periods of reversible immobility, increased arousal thresholds, and robust homeostatic rebound following deprivation, supporting their validity as a vertebrate sleep model.^[^ ^29^, ^30^, ^31^ ^]^ Thus, the observed rebound sleep serves as a reliable proxy for confirming effective sleep loss during the deprivation phase. We observed that zebrafish subjected to 24 h of sleep deprivation exhibited marked rebound sleep behavior during the recovery phase, with significant increases in sleep duration observed during both the light and dark phases (Figure 1J–L). Specifically, the proportion of consolidated sleep episodes decreased to 34.09% in the SD group compared with 61.85% in the control group, accompanied by an elevated sleep fragmentation index (Figure 1M). These results suggest that SD primarily impairs sleep continuity in zebrafish. To extend these findings to a large mammalian model, we implemented a 96‐h SD protocol in pigs, which was monitored under controlled conditions. Video‐based behavioral analyses revealed a pronounced increase in wakefulness, with active states accounting for 88.4% of the observation time, compared with 40.8% under baseline conditions (Figure S1C,D, Supporting Information).

Collectively, these tri‐species models—mice, zebrafish, and pigs—provide a robust framework for investigating the conserved and species‐specific physiological responses to SDs, offering valuable insights into the mechanisms underlying SD‐induced immune dysregulation.

### 2.2 Single‐cell Transcriptomic Profiling Reveals Neutrophil Expansion Following Sleep Deprivation

**Figure 2:**
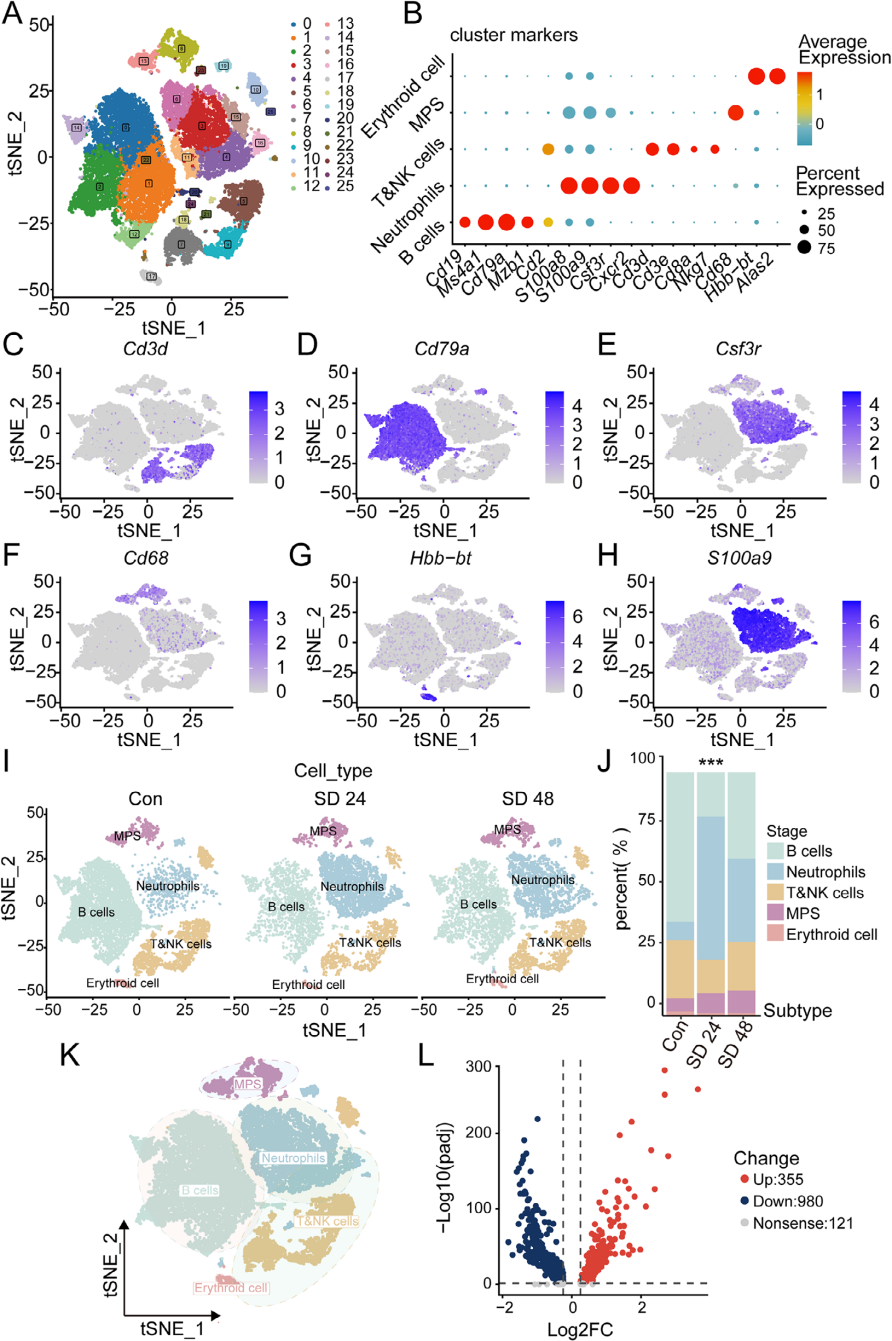
Single‐cell RNA sequencing analysis of immune cells following sleep deprivation. A) t‐SNE plot displaying the clustering of immune cells identified in the dataset, with clusters colored by cell type. Each cluster represents a different immune cell population, and the plot shows how the cells are grouped on the basis of their gene expression profiles. B) Dot plot showing cluster‐specific marker genes for the identified cell types, with each dot representing a marker gene. The color scale indicates the average expression level of the gene, while the size of the dot corresponds to the percentage of cells expressing each marker gene. C–H) Feature plots depicting the expression of specific marker genes for various immune cell types: C) Cd3d (T cells), D) Cd79a (B cells), E) Csf3r (neutrophils), F) Cd68 (monocytes/macrophages, MPS), G) Hbb‐bt (erythroid cells), and H) S100a9 (neutrophils). Each feature plot shows the expression intensity of the respective marker gene across the t‐SNE plot, with colors representing the level of expression. I) t‐SNE plots comparing the distributions of different immune cell types in control (Con) and sleep‐deprived (SD) conditions, with data collected at 24 and 48 h post‐SD. J) Bar plot quantifying the proportions of major immune cell populations (B cells, neutrophils, T/NK cells, MPS, and erythroid cells) under both control and SD conditions. Statistical analysis revealed significant differences in the cell populations between the groups. K) Annotated t‐SNE plot highlighting the major cell types (neutrophils, monocytes/macrophages, B cells, T/NK cells, and erythroid cells) for better visualization and understanding of the cell type composition in the dataset. L) Volcano plot showing differentially expressed genes (DEGs) between the SD and control conditions. Genes whose expression was significantly upregulated are shown in red, those whose expression was downregulated are shown in blue, and genes whose expression was not significantly upregulated are shown in gray. The plot provides an overview of the transcriptional changes in immune cells following sleep deprivation.

To comprehensively characterize the systemic immune response to sleep deprivation (SD), we performed single‐cell RNA sequencing (scRNA‐seq) analysis of peripheral blood cells isolated from C57BL/6J mice under control (Con), 24‐h (SD24), and 48 h (SD48) SD conditions. After stringent quality control filtering and unsupervised clustering, we identified 25 transcriptionally distinct cell clusters (**Figure** **2**A), which were further annotated into five major immune cell types, namely, neutrophils, B cells, T/NK cells, mononuclear phagocyte system (MPS) cells, and erythroid cells, on the basis of the expression of canonical marker genes (Figure 2B). Marker gene expression patterns, such as Cd19 for B cells, S100a8 for neutrophils, and Cd3e for T/NK cells, enabled precise annotation of these clusters (Figure 2C–H, Figure S2A–I, Supporting Information).

SD induced substantial shifts in immune cell composition. The proportion of neutrophils markedly increased after both SD24 and SD48, whereas the percentage of B cells significantly decreased (Figure 2I,J). This shift reflects an SD‐induced proinflammatory cellular milieu, with neutrophils emerging as key mediators of the innate immune response. Neutrophils, as the first responders in innate immunity, exhibit transcriptional activation of pathways involved in cell migration, cytokine production, and inflammatory signaling, indicating a systemic transition toward heightened inflammation.

Differential gene expression analysis revealed 355 upregulated and 980 downregulated genes in neutrophils under SD conditions compared with those in controls (Figure 2K,L). The upregulated genes were enriched predominantly in pathways related to chemotaxis, immune activation, and inflammatory responses, underscoring the pivotal role of neutrophils in SD‐induced immune dysregulation.

Together, our scRNA‐seq data reveal that sleep deprivation induces marked shifts in the circulating immune cell composition, most notably characterized by an increased proportion of neutrophils and reduced B cells. Neutrophils displayed pronounced transcriptional changes, including the activation of genes related to chemotaxis and inflammation, indicating increased innate immune activity. These findings highlight neutrophils as key cellular responders to sleep deprivation and suggest that sleep loss rapidly skews the immune landscape toward a proinflammatory state.

### 2.3 Sleep Deprivation Induces Neutrophil Accumulation and Activation across Species

To investigate the impact of sleep deprivation (SD) on immune responses, we assessed neutrophil counts and the expression of genes related to neutrophil activation in three model organisms: mice, zebrafish, and pigs. Our results demonstrated that SD significantly increased the number of neutrophils across all three species, suggesting a conserved immune response to SD. These findings highlight a consistent pattern of neutrophil accumulation and activation, underscoring a shared immunological mechanism triggered by sleep loss.

**Figure 3:**
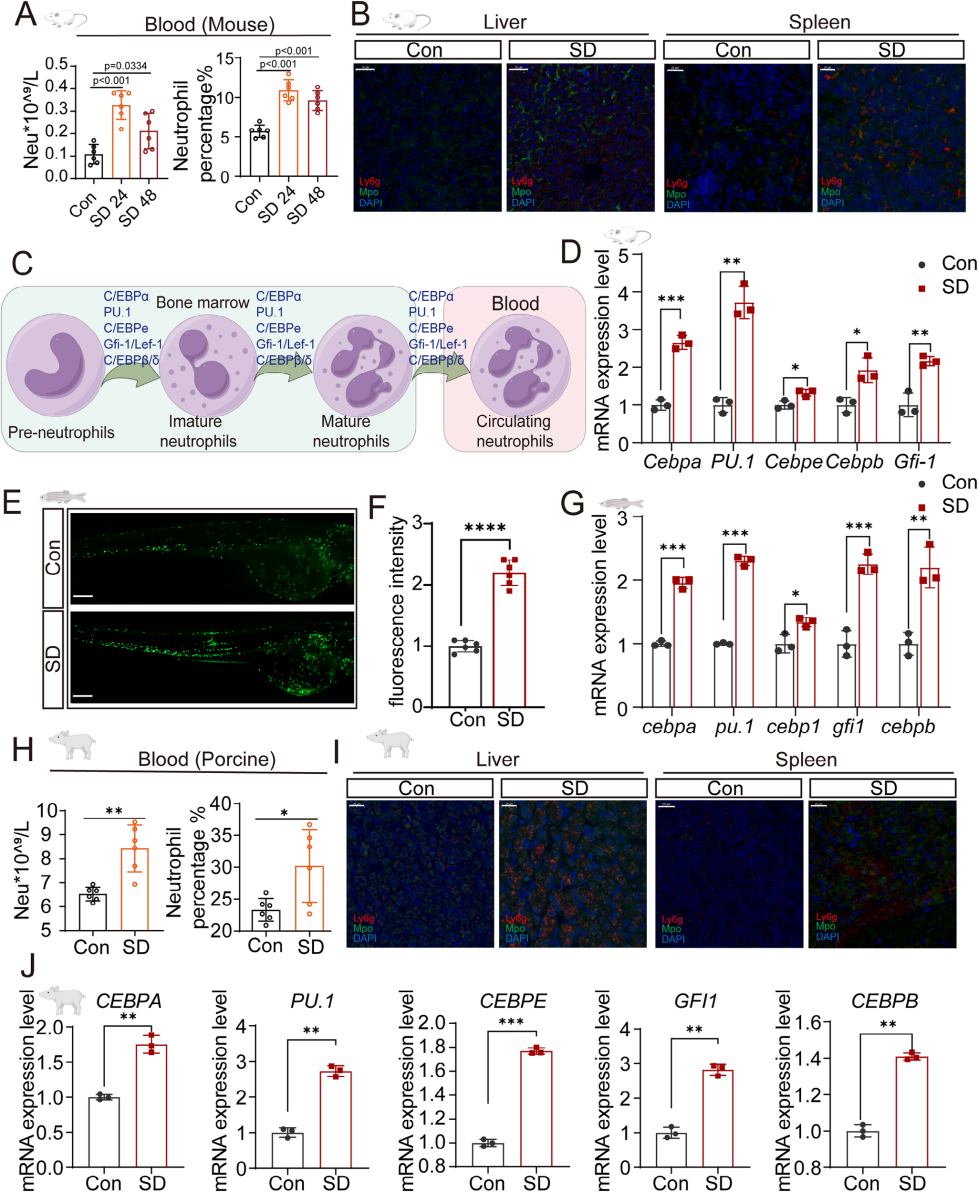
Sleep deprivation **increases the number of neutrophils** in mice, zebrafish, and pigs. A) Quantification of neutrophil counts and percentages in **the** peripheral blood of control and sleep**‐deprived mice** at 24 and 48 h **postSD** (*n* = 6/group). B) Immunofluorescence analysis showing the distribution of neutrophils (Ly6g^+^ and MPO^+^ cells) in the liver and spleen tissues of Con and SD mice at 48 h. Scale bar = 20 µm. Increased neutrophil infiltration was observed in the SD group (*n* = 3/group). C) Schematic representation of neutrophil maturation and differentiation from BW to peripheral circulation, highlighting key transcription factors such as the C/EBP family and PU.1. D) mRNA expression levels of neutrophil‐associated transcription factors (Cebpa, Pu.1, Cebpe, Cebpb, and Gfi1) in neutrophils isolated from the peripheral blood of Con and SD 48 h‐old mice (*n* = 3/group). E) Zebrafish larvae at 4 days postfertilization (dpf) were subjected to sleep deprivation for 24 h. The total number of fluorescently labeled neutrophils in the larvae was assessed under control and SD conditions. Representative fluorescence images show the neutrophil distribution in the larvae. Images were acquired via a fluorescence microscope, and the fluorescence intensity was quantified via ImageJ software (*n* = 6/group). Scale bar: 100 µm. F) Quantification of the fluorescence intensity for neutrophil recruitment in zebrafish, which revealed significantly greater recruitment under SD conditions. G) mRNA expression levels of neutrophil‐related genes in 5 dpf zebrafish larvae under Con and SD conditions. (*n* = 3/group) H) Quantification of neutrophil counts and percentages in the peripheral blood of pigs under Con and SD conditions for 96 h (*n* = 6/group). SD significantly increased neutrophil counts. I) Immunofluorescence analysis of neutrophil distribution (Ly6g^+^ and MPO^+^ cells) in liver and spleen tissues of Con and SD 96 h pigs (*n* = 3/group). Increased neutrophil presence was observed in both liver and spleen tissues under SD conditions. Scale bar = 20 µm. J) mRNA expression levels of neutrophil‐associated transcription factors (CEBPA, PU.1, CEBPE, CEBPB, and GFI1) in neutrophils isolated from the peripheral blood of Con and SD 96 h pigs (*n* = 3/group). Statistical significance was analyzed with unpaired t tests, with significance levels denoted as follows: ns, *p* > 0.05; *, *p* < 0.05; **, *p* < 0.01; ***, *p* < 0.001; ****, *p* < 0.0001.

In mice, SD led to a significant increase in peripheral blood neutrophil counts at both 24 and 48 h postdeprivation, with the most pronounced increase observed at 24 h (**Figure** **3**A). Concurrently, complete blood count analysis revealed a significant increase in total leukocyte counts following SD. Although the percentage of lymphocytes appeared to be reduced, this change was likely due to the relative increase in the number of neutrophils rather than an actual decrease in their absolute numbers (Figure S3A, Supporting Information). Histological and immunofluorescence analyses of liver and spleen tissues revealed extensive neutrophil infiltration, as indicated by Ly6G^+^ and MPO^+^ staining, in the SD mice compared with the controls (Figure 3B; Figure S3C, Supporting Information). Consistent with these observations, the mRNA expression levels of proinflammatory cytokines (*Il‐1*, *Il‐6*, *Il‐8*, and *Tnf‐α*) were markedly increased in the liver and spleen of SD mice (Figure S3D,E, Supporting Information), indicating widespread immune activation. Additionally, transcriptional profiling of blood neutrophils revealed significant upregulation of key transcription factors, including *Cebpa*, *Pu.1*, *Cebpe*, *Cebpb*, and *Gfi1* (Figure 3C,D), suggesting that SD not only promotes neutrophil mobilization but also primes their functional activation.

In zebrafish larvae subjected to 24‐h SD, fluorescence labeling and quantitative analysis demonstrated a significant increase in neutrophil recruitment (Figure 3E,F). Transcriptional analysis further revealed the upregulation of neutrophil‐related genes, including *cebpa*, *pu.1*, *cebpb*, *gfi1*, and *cebpd*, as well as proinflammatory cytokines (*il‐1*, *il‐6*, *cxcl8a*, and *tnf‐α*) (Figure 3G; Figure S3F, Supporting Information), mirroring the inflammatory response observed in mice.

Extending these findings to pigs, we observed a similar increase in peripheral blood neutrophil counts and proportions following 96‐h SD (Figure 3H; Figure S3B, Supporting Information). Immunofluorescence analysis of liver and spleen tissues confirmed substantial neutrophil infiltration in SD pigs (Figure 3I), accompanied by elevated expression of neutrophil‐associated transcription factors (*CEBPA*, *PU.1*, *CEBPB*, *GFI1*, and *CEBPB*) (Figure 3J) and proinflammatory cytokines (*IL‐1*, *IL‐6*, *IL‐8*, and *TNF‐α*) in these tissues (Figure S3H–I, Supporting Information).

Collectively, our multispecies analysis demonstrated that sleep deprivation triggers a conserved immune response characterized by neutrophil accumulation and activation. This process is associated with the upregulation of key transcription factors and proinflammatory pathways, highlighting neutrophils as central mediators in the immune response to sleep deprivation.

### 2.4 Sleep Deprivation Activates Glycolysis and Lactylation in Neutrophils

**Figure 4:**
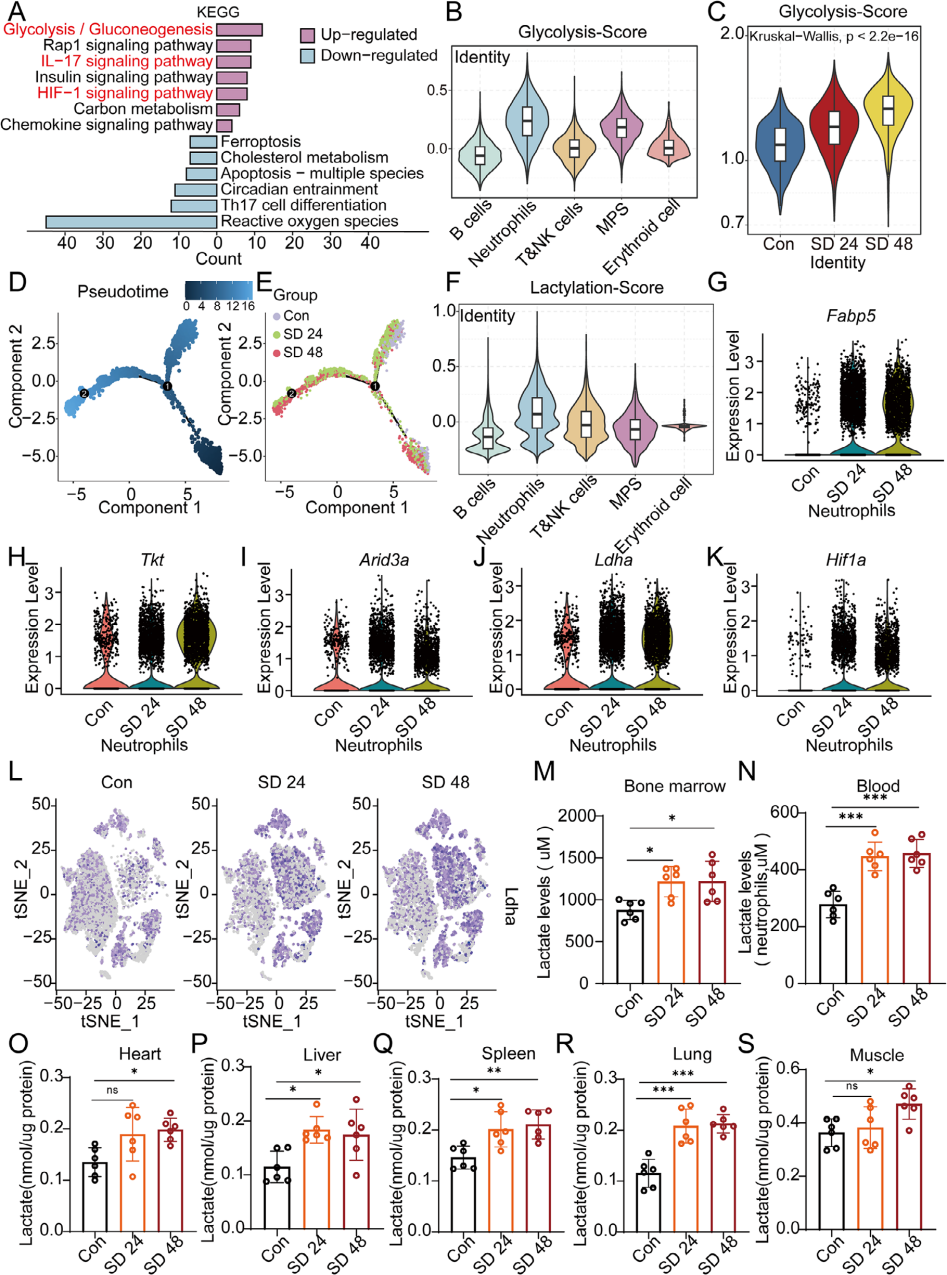
Sleep deprivation induces glycolysis and lactylation in neutrophils. A) KEGG pathway enrichment analysis showing the upregulated and downregulated pathways under SD conditions. The upregulated pathways, including glycolysis/gluconeogenesis and IL‐17 signaling, are highlighted in red, whereas the downregulated pathways are highlighted in blue. B) Glycolysis scores for various immune cell populations, showing the highest glycolysis activity in neutrophils under SD conditions. The data were analyzed via the Kruskal–Wallis test. C) Glycolysis scores specifically for neutrophils under control (Con), SD 24 h, and SD 48 h conditions, with significant differences observed between the groups. Statistical significance was determined via the Kruskal–Wallis test. D,E) Pseudotime analysis illustrating changes in neutrophil transcription over time under SD conditions. Panel D shows the trajectory of neutrophil differentiation, while Panel E compares the transcriptional profiles between the Con, SD 24 h, and SD 48 h groups. The numbers “1” and “2” represent distinct branch points or cell state transitions identified within this inferred trajectory. Branch Points 1 (pseudotime ≈8) and 2 (pseudotime ≈12–16) demarcate key lineage‐splitting events. Early divergence (Branch 1) correlates with treatment conditions (Con/SD 24), whereas late specialization (Branch 2) aligns with prolonged exposure (SD 48). F) Lactylation scores across different immune cell populations, highlighting that neutrophils have the highest level of lactylation. The data were analyzed via the Kruskal‒Wallis test. G–K) Expression levels of glycolysis‐ and lactylation‐associated genes in neutrophils: G) Fabp5, H) Tkt, I) Arid3a, J) Ldha, and K) Hif1a, showing significant upregulation of these genes in neutrophils following SD at both 24 and 48 h. L) t‐SNE plots showing Ldha expression in neutrophils from the Con, SD 24 h, and SD 48 h groups, indicating a shift in Ldha expression with increasing SD. M,N) Lactate levels in neutrophils from BW (M) and blood (N) under Con, SD 24 h, and SD 48 h conditions, showing significant increases in lactate levels in the SD groups. Statistical significance was determined via one‐way ANOVA with Tukey's post hoc test (*n* = 6/group). O‐S) Lactate levels in various tissues (heart, liver, spleen, lung, and muscle) in the Con, SD 24 h, and SD 48 h groups. Significant increases in lactate levels were observed in liver, spleen, and lung tissues following SD, **as** analyzed by one‐way ANOVA with Tukey's post hoc test (*n* = 6/group).

To explore the metabolic changes induced by sleep deprivation (SD) in neutrophils, we first performed Kyoto Encyclopedia of Genes and Genomes (KEGG) pathway enrichment analysis on upregulated genes from circulating neutrophils collected at 24 h (SD24h) and 48 h (SD48h) following SD. The KEGG analysis revealed significant enrichment in metabolic pathways, particularly glycolysis and gluconeogenesis, indicating a shift toward glycolytic metabolism in neutrophils during SD (**Figure** **4**A).

We next assessed glycolysis activity in different immune cell types via scRNA‐seq data. Neutrophils presented the highest glycolysis scores, with significant upregulation observed after 24 and 48 h of SD (Figure 4B,C), suggesting that sleep deprivation triggers glycolytic reprogramming in neutrophils.

To further understand the dynamics of gene expression over time during SD, we performed pseudotemporal analysis of neutrophils. This analysis revealed clear activation of the glycolysis pathway as the duration of SD increased (Figure 4D,E). Among the significantly upregulated genes, *Fabp5, Tkt, Arid3a, Ldha*, and *Hif1a* were identified. These genes not only play essential roles in glycolysis but are also involved in lactate production and subsequent lactylation modifications (Figure 4G–L). These findings suggest a close link between enhanced glycolysis and lactate generation, highlighting that the metabolic shift induced by SDs may contribute to the activation of lactylation, a posttranslational modification mediated by lactate, thereby connecting glycolytic reprogramming with functional changes in neutrophils.

In addition to metabolic changes, we examined the lactylation of neutrophils, which is crucial for their activation. We performed lactylation scoring and found that genes involved in lactylation modification were highly enriched in neutrophils following SD (Figure 4F). These findings suggest that lactate, a byproduct of glycolysis, may serve both as a metabolic output and as a signaling molecule to regulate neutrophil activation and function during SD.

To quantify the metabolic consequences of SD, we measured lactate levels in the bone marrow (BW), blood, and various peripheral tissues of SD mice. Lactate levels were significantly elevated across all the tissues examined, including BW (Figure 4M), blood (Figure 4N), heart (Figure 4O), liver (Figure 4P), spleen (Figure 4Q), lung (Figure 4R), and muscle (Figure 4S) weights. These results highlight the systemic metabolic changes associated with sleep deprivation, with lactate accumulation reflecting increased glycolytic activity.

These findings show that sleep deprivation induces glycolytic reprogramming and lactylation in neutrophils, which may play crucial roles in their activation and the inflammatory response.

### 2.5 Sleep Deprivation Enhances Lactylation and Related Enzyme Expression

Lactate dehydrogenase *(LDHA*), *P300*, and histone deacetylase 3 (*HDAC3*) are key enzymes involved in lactate metabolism and the regulation of lactylation modifications, which play important roles in immune and metabolic responses.^[^ ^32^ ^]^ To investigate how sleep deprivation affects lactylation and its associated enzymatic machinery, we analyzed the expression of these enzymes and their corresponding lactylation modifications across three species: mice, zebrafish, and pigs.

**Figure 5:**
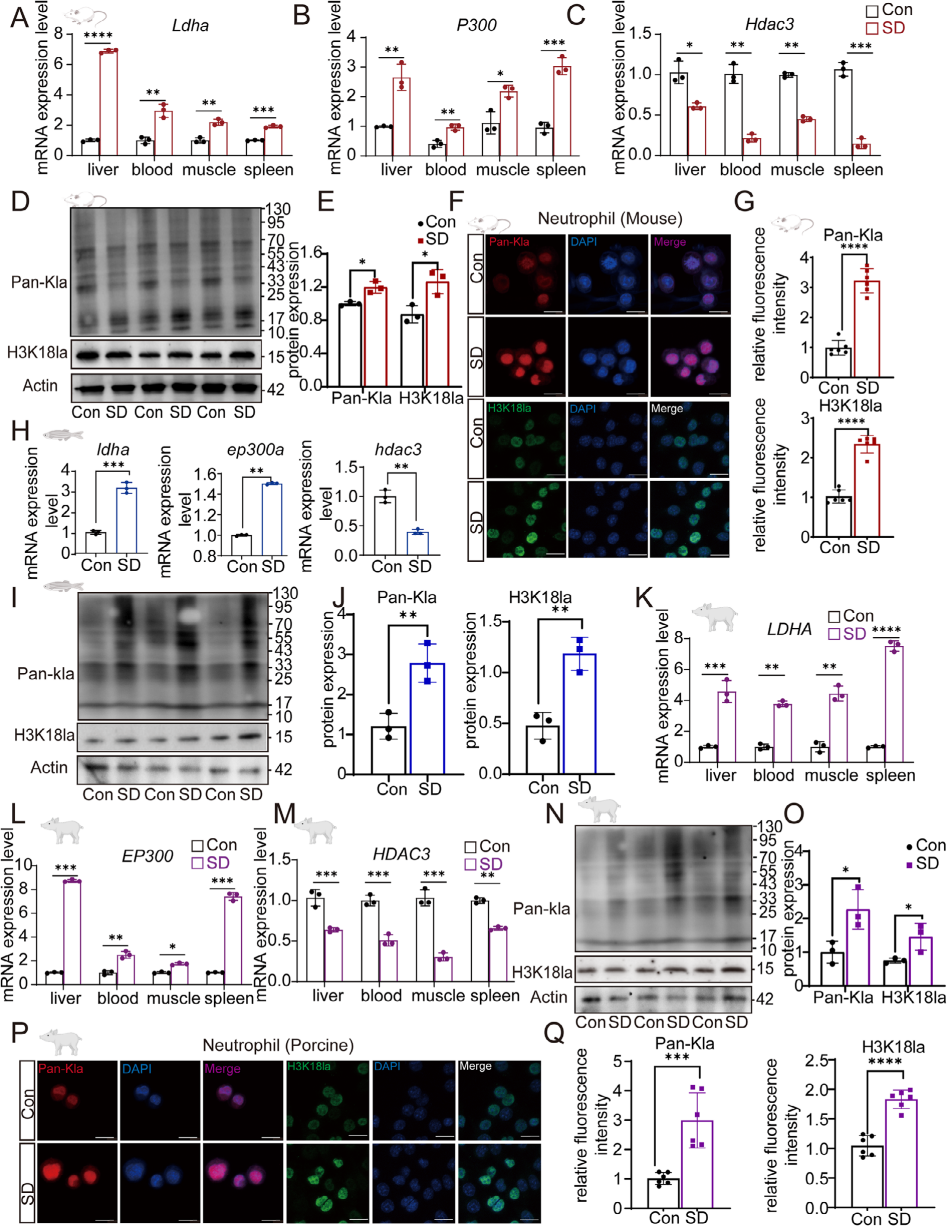
Sleep deprivation (SD) increases lactylation levels in mouse tissues, zebrafish larvae, and pig tissues. A‐C) mRNA expression of Ldha, P300, and Hdac3 in various mouse tissues (liver, blood, muscle, and spleen) under control and sleep‐deprived conditions. (*n* = 3/group). D) Western blot analysis of total lactylation (Pan‐Kla) and histone H3K18 lactylation (H3K18la) in mouse blood neutrophils under Con and SD conditions (*n* = 3/group). Representative bands are shown, with quantification provided in panel E. E) Quantification of Pan‐Kla and H3K18la protein expression levels in mouse blood neutrophils. F) Immunofluorescence images showing Pan‐Kla and H3K18la expression in mouse neutrophils. Scale bar = 20 µm. G) Relative fluorescence intensity of Pan‐Kla and H3K18la in mouse neutrophils, as measured by fluorescence microscopy. H) mRNA expression levels of lactylation‐related genes in zebrafish larvae under Con and SD conditions for 24 h, revealing the upregulation of genes associated with lactylation. I) Western blot analysis of Pan‐Kla and H3K18la in zebrafish, which revealed increased lactylation in response to SDs. J) Quantification of Pan‐Kla and H3K18la protein expression levels in zebrafish larvae. K‒M) mRNA expression of LDHA, EP300, and HDAC3 in pig tissues (liver, blood, muscle, and spleen) under Con and SD conditions (*n* = 3 per group). N) Western blot analysis of Pan‐Kla and H3K18la in pig blood neutrophils (*n* = 3 per group), which revealed increased lactylation in neutrophils following SD. O) Quantification of Pan‐Kla and H3K18la protein levels in pig neutrophils, with significant differences between the Con and SD groups. P) Immunofluorescence images showing Pan‐Kla and H3K18la expression in pig neutrophils. Scale bar = 20 µm. Q) Relative fluorescence intensity of Pan‐Kla and H3K18la in pig neutrophils (*n* = 6/group), showing a significant increase in fluorescence intensity under SD conditions. Statistical significance was analyzed with unpaired t tests, with significance levels denoted as follows: ns, *p* > 0.05; *, *p* < 0.05; **, *p* < 0.01; ***, *p* < 0.001; ****, *p* < 0.0001.

In mice, we assessed the mRNA expression of *Ldha, P300*, and *Hdac3* in multiple tissues, including the liver, blood, muscle, and spleen. As shown in **Figure** **5**A‒C, SD significantly upregulated *Ldha* and *P300* while downregulating Hdac3 in these tissues. Western blot analysis further confirmed a significant increase in total protein lactylation (Pan‐Kla) and H3K18 lactylation (H3K18la) in neutrophils from SD mice (Figure 5D). Quantification revealed markedly elevated levels of Pan‐Kla and H3K18la (Figure 5E). Immunofluorescence imaging corroborated these findings, revealing increased fluorescence intensity of Pan‐Kla and H3K18la in neutrophils from SD mice (Figure 5F,G).

Next, we examined zebrafish larvae subjected to 24 h of SD. Like those in the mouse model, the mRNA expression levels of lactylation‐related genes, including *ldha, ep300a*, and *hdac3*, were significantly altered, with ldha and ep300a being upregulated and hdac3 being downregulated following SD (Figure 5H). Western blot analysis of zebrafish proteins confirmed higher levels of Pan‐Kla and H3K18la in SD larvae than in control larvae (Figure 5I,J).

In pigs, consistent trends were observed, with significant changes in the mRNA expression of LDHA, EP300, and HDAC3 in the liver, blood, muscle, and spleen (Figure 5K–M). SD resulted in upregulation of *LDHA* and *EP300* and downregulation of *HDAC3*. Western blot analysis of neutrophils isolated from SD pigs revealed increased levels of Pan‐Kla and H3K18la (Figure 5N,O). These results were further validated by immunofluorescence imaging, which revealed increased fluorescence intensity of Pan‐Kla and H3K18la in SD pig neutrophils (Figure 5P,Q).

These results suggest that sleep deprivation activates lactylation and its associated enzymes, which may serve as a conserved mechanism for immune and metabolic adaptation.

### 2.6 Exogenous Lactate Enhances Lactylation and Neutrophil Activation

To investigate the immunoregulatory role of lactate, we examined the effects of exogenous lactate administration on lactylation and neutrophil activation in mice and zebrafish.

**Figure 6:**
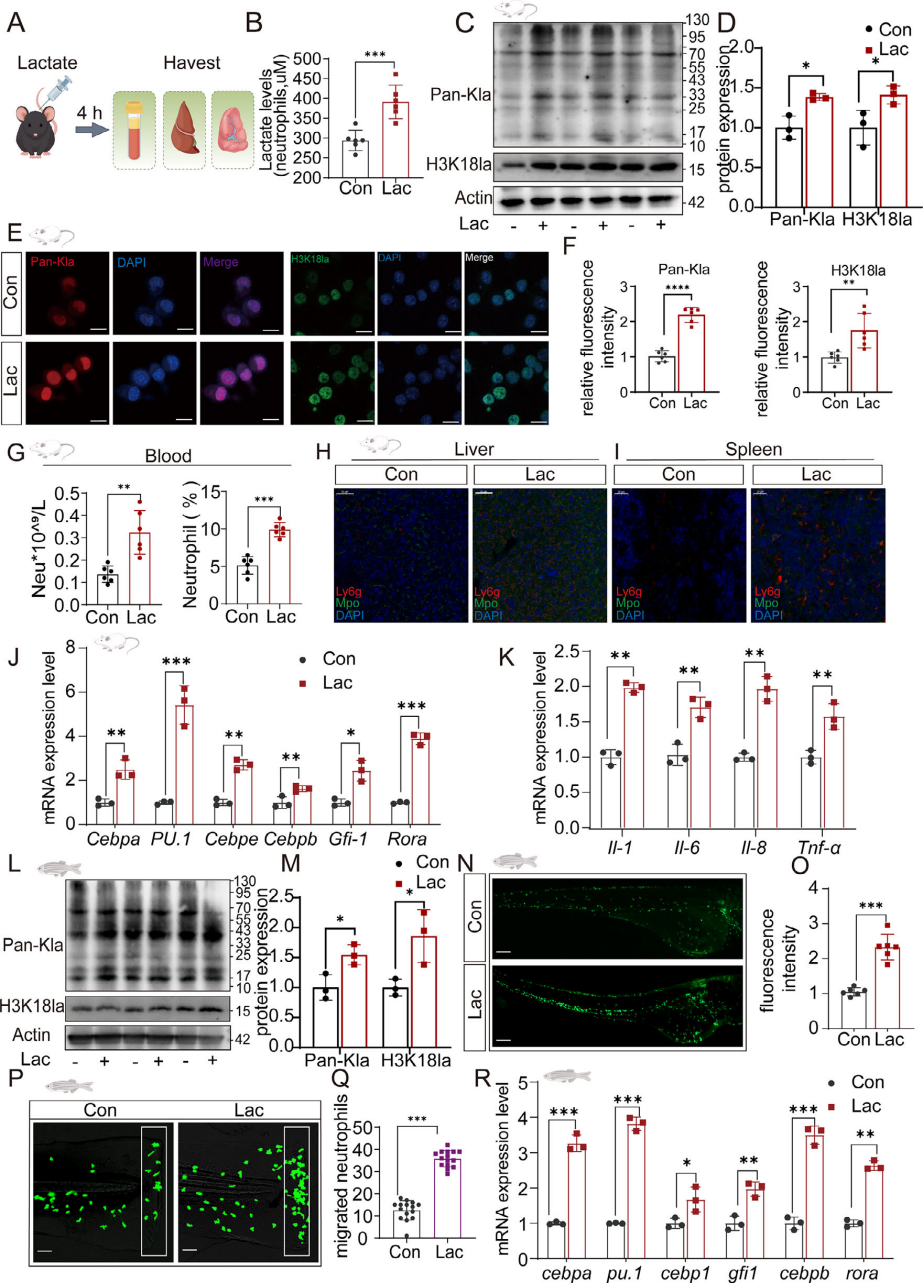
Lactate enhances neutrophil lactylation and gene expression levels in vivo. A) Schematic diagram illustrating the experimental procedure in which lactate (50.5 mg kg^−1^ body weight) was administered in PBS, followed by tissue sampling (liver, blood, and spleen) four h postinjection. B) Measurement of lactate levels in neutrophils isolated from the blood of mice treated with lactate (Lac) or PBS (Con). (*n* = 6/group). C) Western blot analysis of total lactylation (Pan‐Kla) and histone H3K18 lactylation (H3K18la) protein levels in blood neutrophils isolated from mice treated with either PBS (Con) or lactate (Lac). D) Quantification of Pan‐Kla and H3K18la protein expression levels in blood neutrophils. E) Immunofluorescence images showing Pan‐Kla and H3K18la expression in mouse neutrophils. Scale bar = 20 µm. F) Quantification of the relative fluorescence intensity of Pan‐Kla and H3K18la in neutrophils from the Con and Lac groups (*n* = 6/group). G) Neutrophil counts and percentages in blood following lactate treatment (*n* = 6/group). H, I) Representative immunofluorescence images of neutrophils (Ly6G^+^ and Mpo^+^) in the liver (H), scale bar = 50 µm, and spleen (I) tissue sections from Con and Lac mice (*n* = 3 per group), scale bar = 20 µm. J) Gene expression levels of neutrophil‐associated transcription factors (Cebpa, PU.1, Cebpe, Cebpb, Gfi1, and Rora) in blood neutrophils. K) Gene expression levels of proinflammatory cytokines (Il‐1, Il‐6, Il‐8, and Tnf‐α) in blood neutrophils. L) Western blot analysis of Pan‐Kla and H3K18la in zebrafish larvae under control (Con) and lactate (Lac) conditions (*n* = 3/group), revealing increased lactylation in zebrafish larvae following lactate treatment. M) Quantification of protein expression levels (Pan‐Kla and H3K18la) in zebrafish larvae. N) Zebrafish larvae at 5 days postfertilization (dpf) were treated with lactate for 4 h. The total number of fluorescently labeled neutrophils in the larvae. O) Representative fluorescence images showing the neutrophil distribution in the larvae (*n* = 6 per group). Scale bar: 100 µm. P) Representative images of neutrophil migration during caudal fin damage in zebrafish larvae, with increased migration observed following lactate treatment (*n* = 15/group). Q) Quantification of migrated neutrophils in zebrafish larvae, showing significant increases in migration following lactate treatment. R) Gene expression levels of transcription factors (Cebpa, PU.1, Cebp1, Gfi1, Cebpb, and Rora) in zebrafish larvae. Statistical significance was analyzed with unpaired t tests, with significance levels denoted as follows: ns, *p* > 0.05; *, *p* < 0.05; **, *p* < 0.01; ***, *p* < 0.001; ****, *p* < 0.0001.

In mice, tissue samples were collected 4 h after the intraperitoneal injection of lactate (**Figure** **6**A). Lactate administration significantly elevated blood lactate levels (Figure 6B). The expression of genes related to lactate metabolism and lactylation was assessed. Lactate treatment notably upregulated Ldha, while Ep300, a histone acetyltransferase, increased significantly, and Hdac3, a histone deacetylase, was downregulated (**Figure** S4A–C, Supporting Information). Western blot analysis confirmed a marked increase in global lactylation (Pan‐Kla) and H3K18 lactylation (H3K18la) in neutrophils (Figure 6C). Immunofluorescence analysis revealed greater fluorescence intensities of Pan‐Kla and H3K18la in neutrophils from lactate‐treated mice than in those from control mice (Figure 6E,F).

Neutrophil counts and percentages in the peripheral blood of lactate‐treated mice revealed significant recruitment (Figure 6G). Immunofluorescence staining revealed increased neutrophil infiltration in the liver and spleen following lactate treatment (Figure 6H–I). Additionally, the mRNA expression of the neutrophil‐associated transcription factors *Cebpa, PU.1, Cebpe, Cebpb, Gfi1*, and *Rora* was significantly upregulated in neutrophils isolated from lactate‐treated mice (Figure 6J). Proinflammatory cytokines, including *Il‐1, Il‐6, Il‐8*, and *Tnf‐α*, were also elevated in the blood of the lactate‐treated mice (Figure 6K).

To further extend these findings, we employed zebrafish larvae as an in vivo model. Lactate administration significantly increased the expression of genes related to lactate metabolism and lactylation, such as *ldha* and *ep300a*, while reducing *hdac3* expression (Figure S4D–F, Supporting Information). Western blot analysis confirmed elevated Pan‐Kla and H3K18la levels in lactate‐treated zebrafish (Figure 6L–M). Fluorescence imaging revealed increased neutrophil fluorescence intensity in lactate‐treated zebrafish (Figure 6N–O). Notably, lactate significantly enhanced neutrophil migration in the zebrafish caudal fin injury model (Figure 6P–Q). Furthermore, the mRNA expression of neutrophil‐specific transcription factors (*cebpa, pu.1, cebpb, gfi1*, and *rora*) and proinflammatory cytokines (*il‐1, il‐6, cxcl8a*, and *tnf‐α*) was significantly increased in the lactate‐treated zebrafish (Figure 6R).

These findings demonstrate that exogenous lactate administration enhances lactylation and neutrophil activation in both mice and zebrafish. The upregulation of lactylation‐related enzymes, neutrophil‐specific transcription factors, and proinflammatory cytokines highlights the critical role of lactate in immune modulation.

### 2.7 Inhibition of Glycolysis and Lactate Production Attenuates SD‐Induced Neutrophil Activation

**Figure 7:**
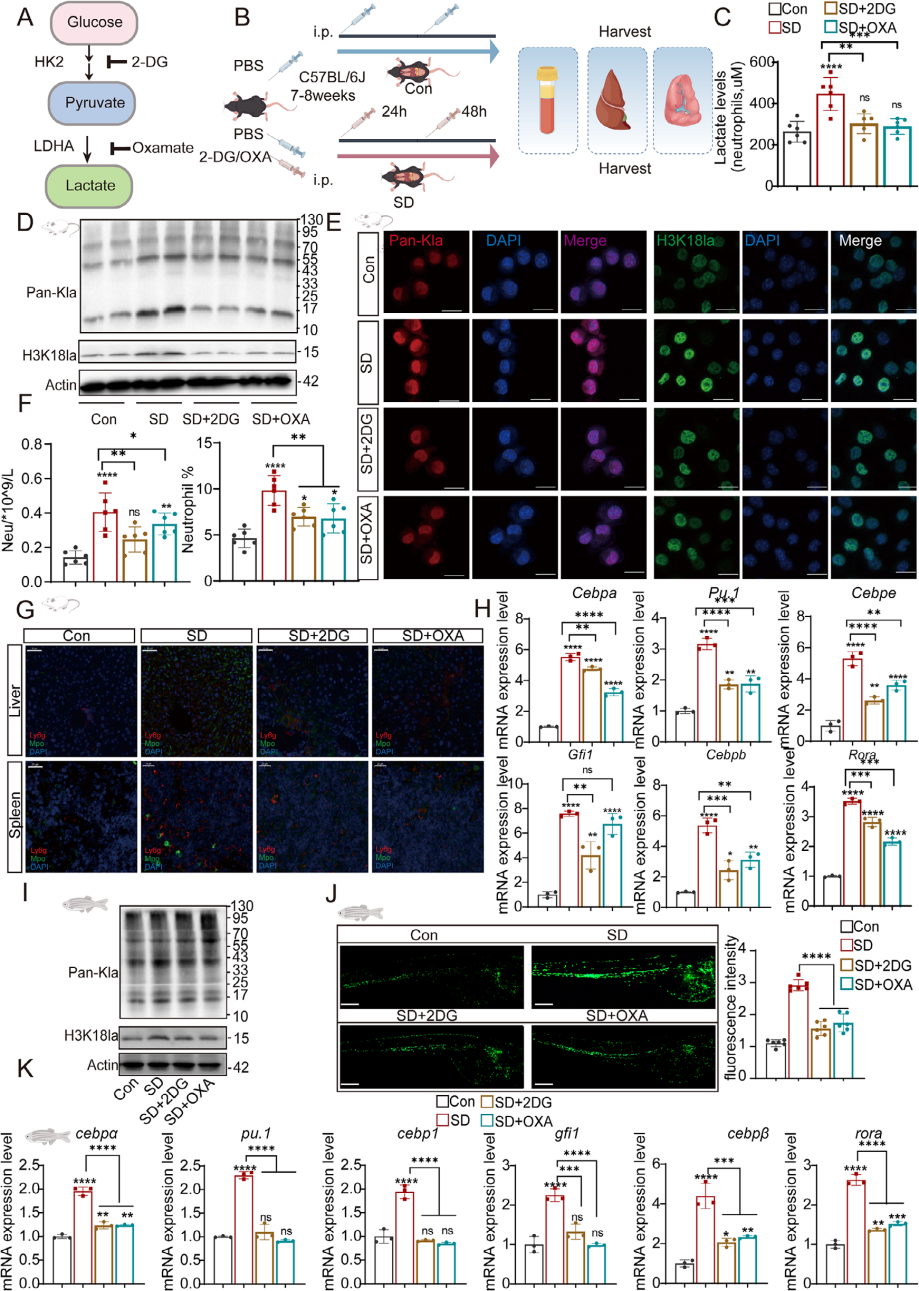
Lactylation modification and its impact on neutrophil‐related changes in mice and zebrafish following 2DG and OXA intervention. A) Schematic representation of the metabolic pathway involved in glucose metabolism, where 2DG inhibits hexokinase (HK) activity and OXA inhibits LDHA, leading to the modulation of lactate levels. B) Experimental design in which C57BL/6J mice were used to investigate the effects of SD and treatment with 2DG and OXA. The mice were injected with PBS or 2DG/OXA 3 h before the onset of sleep deprivation and again 24 h after SD initiation. Tissues were harvested for analysis after 48 h of continuous sleep deprivation. C) Lactate levels in blood neutrophils isolated from the different treatment groups (Con, SD, SD + 2DG, and SD + OXA). D) Western blot analysis showing Pan‐Kla and H3K18la expression in mouse tissues, indicating changes in lactylation following treatment (*n* = 3/group). E) Immunofluorescence images showing Pan‐Kla and H3K18la expression in neutrophils from each group, visualized under a fluorescence microscope. Scale bar = 20 µm. (*n* = 6/group). F) Quantification of peripheral blood neutrophil counts under different treatment conditions. Compared with the control, SD led to a significant increase in neutrophil counts. Although the neutrophil counts in the SD + 2‐DG and SD + OXA groups were slightly greater than those in the control group were, both treatments significantly attenuated the increase observed in the SD group (*n* = 6/group). G) Immunofluorescence analysis showing the distribution of neutrophils (Ly6g^+^ and MPO^+^ cells) in the liver and spleen tissues of different treatment groups (Con, SD, SD + 2DG, and SD + OXA) in mice. Scale bar = 20 µm. H) mRNA expression levels of neutrophil‐related genes (Cebpa, Pu.1, Cebpe, Gfi‐1, Cebpb, and Rora) in mouse neutrophils, revealing significant upregulation of neutrophil‐related genes in the SD and SD + 2DG/OXA groups compared with the control group. I) Western blot analysis of Pan‐Kla and H3K18la protein expression levels in zebrafish from each treatment group, showing increased lactylation modifications in response to SDs and intervention. J) Zebrafish larvae at 5 days postfertilization were subjected to sleep deprivation (SD) for 24 h, with additional treatments of 2DG and OXA. The total number of fluorescently labeled neutrophils in larvae was measured under four conditions: control (Con), sleep deprivation (SD), SD + 2DG, and SD + OXA. Representative fluorescence images show the neutrophil distribution in the larvae. Images were acquired via a fluorescence microscope, and the fluorescence intensity was quantified via ImageJ software (*n* = 6/group). Scale bar: 100 µm. K) mRNA expression analysis of neutrophil‐associated genes (cebpα, pu.1, cebpβ, gfi1, and rora) in zebrafish following treatment with lactate‐modifying agents (2DG/OXA). Significant changes in gene expression were observed in the treated groups compared with the control groups. The data were analyzed via one‐way ANOVA with Tukey's post hoc test, with significance levels denoted as follows: ns, *p* > 0.05; *, *p* < 0.05; **, *p* < 0.01; ***, *p* < 0.001; ****, *p* < 0.0001.

To investigate the role of glycolysis and lactate metabolism in neutrophil lactylation and activation induced by sleep deprivation (SD), we employed 2‐deoxy‐D‐glucose (2‐DG) and oxamate (OXA) to inhibit glycolysis and lactate production, respectively (**Figure** **7**A,B). SD significantly elevated the serum lactate level, which was markedly reduced following 2‐DG and OXA treatment (Figure 7C). Western blot analysis revealed that SD induced a substantial increase in total protein lactylation (Pan‐Kla) and histone H3K18 lactylation (H3K18la) in neutrophils, whereas both 2‐DG and OXA effectively attenuated these modifications (Figure 7D; Figure S5A, Supporting Information). Immunofluorescence staining further confirmed that Pan‐Kla and H3K18la signals were reduced in neutrophils from treated mice (Figure 7E; Figure S5B, Supporting Information).

We next assessed the effects of glycolysis and lactate metabolism inhibition on neutrophil recruitment and inflammation. SD resulted in a significant increase in peripheral blood neutrophil counts and percentages, both of which were attenuated following 2‐DG and OXA treatments (Figure 7F; Figure S5D, Supporting Information). Immunofluorescence analysis of liver and spleen tissues revealed reduced neutrophil infiltration (Ly6G^+^ and MPO^+^ staining) after metabolic intervention (Figure 7G).

At the transcriptional level, SD upregulated the expression of neutrophil‐specific transcription factors, including *Cebpa*, *PU.1*, *Cebpb*, *Cebpe*, *Gfi1*, and *Rora*, suggesting increased neutrophil activation. This upregulation was significantly mitigated by both 2‐DG and OXA treatments (Figure 7H). In addition, proinflammatory cytokines such as *Il‐1*, *Il‐6*, *Il‐8*, and *Tnf‐α* were highly expressed under SD conditions, while their expression levels were markedly reduced upon metabolic inhibition (Figure S5C, Supporting Information).

To validate these observations in vivo, we utilized a zebrafish model. Western blot analysis of zebrafish larvae revealed that SD significantly increased Pan‐Kla and H3K18la levels, which were notably reduced after treatment with 2‐DG and OXA (Figure 7I). Additionally, whole‐body immunofluorescence imaging revealed a substantial increase in neutrophil accumulation throughout the zebrafish body after SD, while both 2‐DG and OXA treatments significantly diminished neutrophil fluorescence intensity, indicating reduced neutrophil activation and recruitment (Figure 7J). In the caudal fin injury model, SD significantly increased neutrophil recruitment, which was substantially diminished following treatment with 2‐DG and OXA (Figure S5D–E, Supporting Information). The expression of neutrophil‐related genes, including *cebpa*, *pu.1*, *cebpb*, *gfi1*, and *rora*, was elevated in SD zebrafish but significantly reduced with metabolic inhibition (Figure 7K). Similarly, the SD‐induced upregulation of proinflammatory cytokines (*il‐1*, *il‐6*, *cxcl8a*, and *tnf‐α*) was effectively suppressed by 2‐DG and OXA treatment (Figure S5F, Supporting Information).

Together, these results highlight the critical role of glycolysis and lactate production in SD‐induced neutrophil activation and inflammation. Inhibiting these metabolic pathways through 2‐DG and OXA not only reduces lactylation modifications but also attenuates neutrophil recruitment and inflammatory cytokine production, providing potential therapeutic targets for mitigating SD‐associated immune dysregulation.

### 2.8 Cut&Tag Analysis Reveals H3K18 Lactylation‐Mediated Rorα Activation in Sleep Deprivation

**Figure 8:**
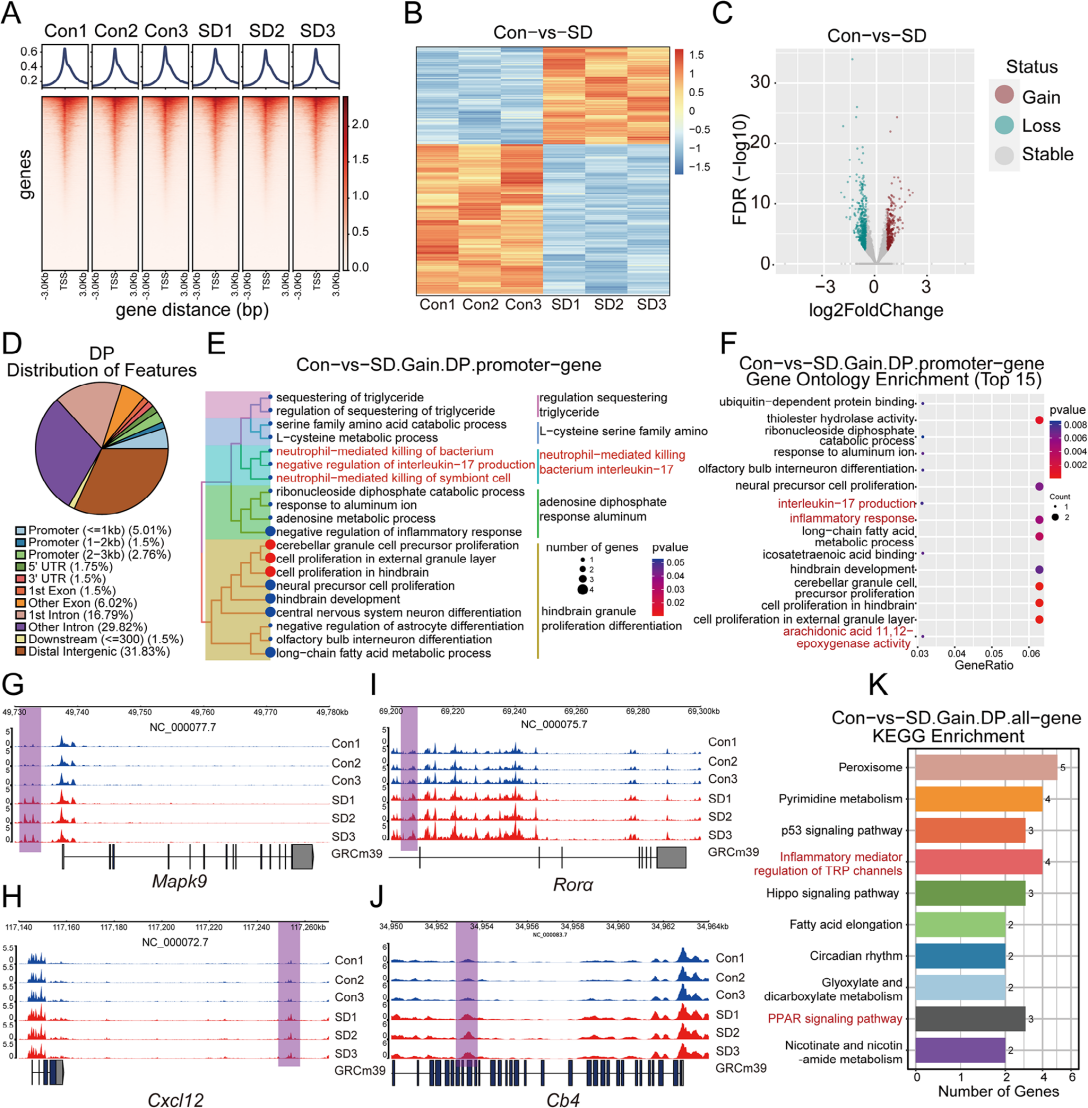
CUT&Tag genome‐wide analysis of the transcriptional effects of H3K18la in response to sleep deprivation. A) Schematic of the experimental design illustrating the comparison between the control and sleep deprivation conditions. Three biological replicates were performed for each group, and CUT&Tag sequencing was conducted to analyze H3K18 lactylation binding at gene promoters. B) Heatmap showing differential H3K18 lactylation binding peaks at genes in response to sleep deprivation, as assessed by CUT&Tag. The heatmap illustrates changes in lactylation modifications between the Con and SD groups. C) Volcano plot highlighting the differentially expressed genes (DEGs) between Con and SD conditions, with genes categorized on the basis of their lactylation status (gain, loss, stable). The plot shows significant changes in gene expression associated with H3K18 lactylation following sleep deprivation. D) Distribution of genomic features associated with differentially regulated promoters following sleep deprivation, indicating the genomic regions most affected by lactylation modification. E) Gene Ontology (GO) enrichment analysis of genes associated with upregulated H3K18 lactylation peaks located in promoter regions, highlighting key immune‐related processes such as neutrophil‐mediated bacterial killing and interleukin‐17 signaling. The enriched GO terms are clustered and summarized according to their positions in the GO hierarchical classification system. The closer the terms are, the more similar their functions are. The results of term induction are presented on the right side; The size of the dots represents the number of genes located in the term in the gene set to be enriched. The color ranges from blue to red, and the significance gradually increases. F) Bubble plot visualization of enriched GO terms reveals significant associations of these genes whose expression is upregulated with immune responses and inflammatory activation, processes closely linked to sleep deprivation. G–J) Genome browser tracks of representative genes (Mapk9, Rora, Cxcl12, and Cb4) showing differential H3K18 lactylation patterns at promoter regions between the control and SD groups, with shaded areas indicating the differential peak regions. K) KEGG pathway enrichment analysis of genes linked to upregulated H3K18 lactylation peaks revealed their involvement in key immune regulatory pathways, including inflammatory mediator regulation of TRP channels, circadian rhythm, and the PPAR signaling pathway. These findings suggest that sleep deprivation modulates the epigenetic regulation of immune function through the dynamic remodeling of H3K18 lactylation.

To elucidate the role of histone H3K18la in SD‐induced neutrophil activation and inflammation, we performed CUT&Tag sequencing targeting H3K18la to assess its impact on the regulation of gene transcription. Heatmaps of H3K18la signals around transcription start sites (TSSs) revealed a pronounced enrichment of H3K18la in the SD group compared with the control group, particularly at promoter regions (**Figure** **8**A). This enrichment correlated with upregulated gene expression, as indicated by differential expression heatmaps and volcano plots (Figure 8B,C), highlighting the regulatory role of H3K18la in gene transcription under SD conditions.

Genomic distribution analysis revealed that H3K18la peaks were distributed across various genomic regions, with a notable proportion localized in promoter regions, as well as substantial enrichment in intronic and intergenic regions (Figure 8D). The functional clustering of genes associated with H3K18la‐enriched promoters revealed significant involvement in biological processes related to the immune response, neutrophil activation, inflammatory regulation, and cellular metabolism (Figure 8E).

GO enrichment analysis of genes with differential H3K18la promoter binding in the SD group revealed strong enrichment in pathways related to neutrophil‐mediated immunity, interleukin‐17 (IL‐17) signaling, and inflammatory responses (Figure 8F). These findings suggest that SD‐induced H3K18la modifications may play a critical role in promoting inflammatory gene expression.

Further examination of SD‐upregulated genes associated with inflammation, immune regulation, and hematopoiesis revealed that *Rorα* exhibited significant H3K18la enrichment at its promoter region, indicating direct transcriptional regulation via lactylation (Figure 8I). In contrast, other upregulated genes, such as *Mapk9*, *Cxcl12*, and *Cb4*, presented H3K18la binding primarily in intronic or distal intergenic regions (Figure 8G,H,J), suggesting indirect regulatory mechanisms. Notably, *Rorα* has been reported to promote neutrophil migration during inflammatory responses, and its direct activation by H3K18la in this study highlights its pivotal role in SD‐induced neutrophil overactivation and systemic inflammation, linking histone lactylation to immune dysregulation. KEGG pathway enrichment analysis revealed that genes with H3K18la‐enriched promoters were significantly involved in metabolic and inflammatory pathways, including pyrimidine metabolism, inflammatory mediator regulation of TRP channels, circadian rhythm, and PPAR signaling (Figure 8K).

Collectively, these results indicate that sleep deprivation enhances H3K18 lactylation, particularly at the promoter region of *Rorα*, leading to its transcriptional activation. This, in turn, promotes the expression of neutrophil‐related functional genes, thereby amplifying neutrophil recruitment and activation and contributing to systemic inflammatory responses. These findings provide mechanistic insights into how histone lactylation modifications regulate SD‐induced immune dysfunction and highlight potential targets for epigenetic interventions to mitigate inflammation‐associated pathologies.

### 2.9 RORα Participates in Neutrophil Migration and Inflammatory Responses During Sleep Deprivation

To further delineate the functional role of RORα in neutrophil migration and inflammatory responses, we employed a zebrafish model with rora gene knockout (*rora* ^−/−^) generated via CRISPR/Cas9. Sleep deprivation (SD), lactate (Lac) treatment, and a caudal fin amputation assay were applied to both wild‐type (*rora* ^+/+^) and *rora* ^−/−^ zebrafish larvae. Neutrophils were visualized via the Tg(mpx:GFP) transgenic line to monitor recruitment in vivo.

**Figure 9:**
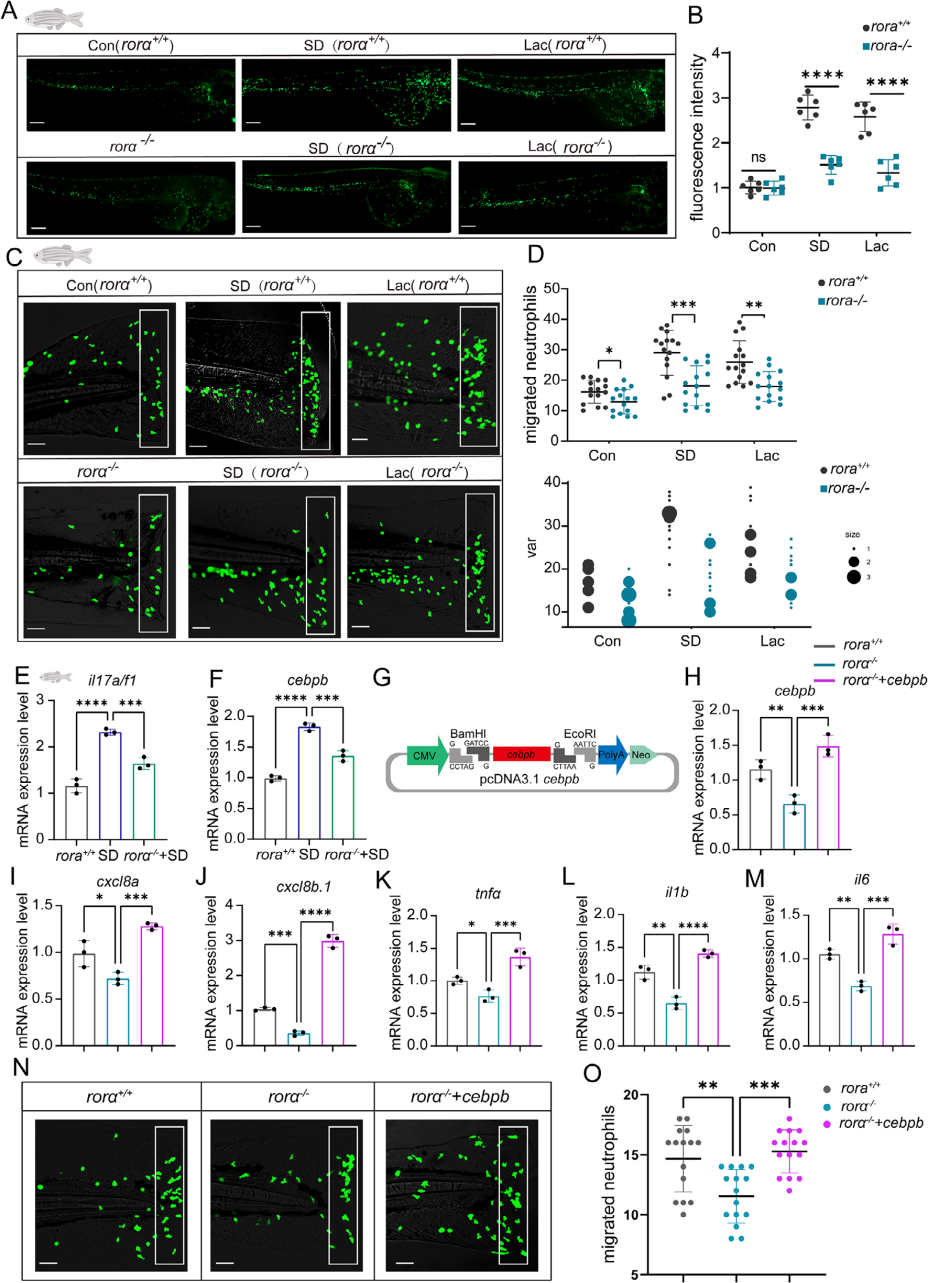
RORα participates in neutrophil migration and inflammatory responses during sleep deprivation. A) Representative fluorescence images of neutrophil distribution in rora^+/+^ and rora^−/−^ zebrafish larvae under control, SD 24 h, and lactate treatment conditions (Lac, 4 h). Neutrophils were visualized in 5 dpf Tg(mpx:GFP) zebrafish via fluorescence microscopy. Scale bar: 100 µm. B) Quantification of the total fluorescence intensity of neutrophils in each group (*n* = 6 per group). C) Representative images showing neutrophil recruitment to the injury site in a caudal fin transection model. Neutrophil migration was significantly enhanced under SD and Lac conditions in rora^+/+^ zebrafish but not in rora^−/^^−^ zebrafish. Scale bar: 100 µm. D) Quantification of migrated neutrophils (top) and variance analysis (bottom) under different conditions (*n* = 15/group). E,F) RT‒qPCR analysis showing that SD induces cebpb expression in WT zebrafish but not in rora^−/−^ mutants. (*n* = 3). G) Schematic of the cebpb overexpression construct (pcDNA3.1‐cebpb). H–M) Overexpression of cebpb in rora^−/−^ zebrafish partially rescued the expression of key inflammatory genes, including cebpb, cxcl8a, cxcl8b.1, tnfa, il1b, and il6, under SD conditions (*n* = 3 per group). N) Representative images of neutrophil migration in WT, rora^−/−^, and rora^−/−^ + cebpb zebrafish following SD and tail injury. Cebpb overexpression restored the impaired migration phenotype in rora‐deficient zebrafish. O) Quantification of migrated neutrophils in each group (*n* = 15/group). Statistical significance was determined **via** unpaired t tests or one‐way ANOVA followed by Tukey's post hoc test, as appropriate. ns, *p* > 0.05; *p < 0.05; **p < 0.01; ***p < 0.001; ****p < 0.0001.

Under control conditions, neutrophils in *rora* ^+/+^ larvae efficiently migrated to the injury site, whereas *rora* ^−/−^ larvae exhibited markedly impaired recruitment (**Figure** **9**A–D). SD and exogenous lactate significantly enhanced neutrophil migration in wild‐type larvae, but this effect was abolished in rora‐deficient fish, indicating that RORα is required for neutrophil mobilization in response to both physical injury and metabolic stress.

To uncover the underlying mechanism, we performed RT‒qPCR analysis. The SD‐induced expression of *il17a/f1* and *cebpb* was significantly lower in the *rora* ^−/−^ zebrafish than in the wild‐type controls (Figure 9E,F). Notably, *cebpb*, a transcription factor central to neutrophil maturation and inflammatory gene expression, was strongly suppressed in the absence of RORα, suggesting a potential regulatory link between these factors.

To test this hypothesis, we constructed a *cebpb* overexpression vector, as C/EBPβ (CCAAT/enhancer‐binding protein beta) is a transcription factor critically involved in myeloid cell differentiation, inflammatory gene regulation, and innate immune responses.^[^ ^33^ ^]^ The construct was microinjected into *rora* ^−/−^ zebrafish embryos (Figure 9G). The overexpression of *cebpb* partially rescued the expression of downstream proinflammatory genes, including *cxcl8a, cxcl8b.1, tnfa, il1b*, and *il6* (Figure 9H–M). Functionally, *cebpb* overexpression also restored neutrophil migration to the injury site in *rora* ^−/−^ larvae (Figure 9N–O), confirming that RORα promotes neutrophil migration at least in part through a *cebpb*‐mediated transcriptional program.

Together, these findings identify RORα as a critical regulator of neutrophil activation and migration under conditions of sleep deprivation and lactate‐induced inflammatory stress. Mechanistically, RORα upregulates *cebpb*, which in turn promotes the expression of key inflammatory mediators necessary for effective neutrophil recruitment.

## 3 Discussion

This study provides novel insights into the intricate relationships among sleep deprivation, lactate metabolism, and immune responses, with a particular emphasis on the role of histone H3K18 lactylation modifications in immune regulation. We demonstrate that acute sleep deprivation significantly alters lactate metabolism, leading to elevated lactate levels in immune cells, especially neutrophils. This metabolic shift drives a series of immune responses, including increased neutrophil recruitment and activation, ultimately triggering a systemic immune response. These findings highlight the pivotal role of lactate metabolism in regulating immune function and underscore the complex interplay between metabolic pathways and immune activation during sleep deprivation.

Lactate metabolism has garnered increasing attention because of its critical involvement in immune regulation,^[^ ^34^ ^]^ particularly during inflammation and infection.^[^ ^35^ ^]^ Lactate has traditionally been considered a mere byproduct of anaerobic metabolism. However, emerging research has revealed its active role in modulating immune cell function. Lactate can influence immune cell polarization, as observed in tumor immunity, where it promotes the M2 polarization of tumor‐associated macrophages (TAMs) through pathways such as ERK and STAT3 activation.^[^ ^36^, ^37^ ^]^ In this context, lactate not only serves as an energy source but also directly affects gene expression through lactylation, a posttranslational modification of histones.^[^ ^21^ ^]^ To further strengthen the specificity of the effects of lactate, we included additional control groups treated with D‐lactate (D‐Lac), an inactive stereoisomer. Only L‐lactate significantly increased the serum lactate level and upregulated the expression of genes associated with lactate metabolism and lactylation (Ldha, Ep300, Hdac3), whereas D‐Lac had no such effect (Figure S4A,B, Supporting Information). Consistently, Western blot analysis revealed robust induction of global protein lactylation (Pan‐Kla) and H3K18 lactylation (H3K18la) following L‐Lac treatment but not following D‐Lac treatment (Figure S4C–E, Supporting Information). Moreover, the expression of proinflammatory cytokines (il‐1, il‐6, cxcl8a, and tnf‐α) and neutrophil migration in the caudal fin injury model were significantly elevated only in the L‐Lac group, supporting a stereospecific and biologically relevant effect of lactate (Figure S4F–H, Supporting Information). These results validate the specificity of the lactate‐induced immunomodulatory response and exclude nonspecific metabolic stress as a confounding factor.

Histone lactylation has recently emerged as a key epigenetic mechanism that regulates immune responses, particularly in inflammatory conditions.^[^ ^38^, ^39^, ^40^ ^]^ Our study provides compelling evidence that lactate accumulation, driven by sleep deprivation, induces histone H3K18 lactylation, which in turn enhances the transcriptional activity of immune‐related genes, thereby amplifying immune responses.

One of the key findings of our study is that sleep deprivation markedly increases histone lactylation in neutrophils. As essential effector cells of the innate immune system, neutrophils rely on metabolic reprogramming to execute their immune functions.^[^ ^41^, ^42^ ^]^ Our results demonstrate that sleep deprivation significantly enhances glycolytic activity in neutrophils, leading to increased lactate production, which in turn promotes histone lactylation—particularly at the promoter region of the Rorα gene.

Retinoic acid receptor‐related orphan receptor alpha (RORA) is a broadly expressed transcription factor belonging to the nuclear hormone receptor superfamily.^[^ ^43^ ^]^ It binds to hormone response elements (HREs) as monomers or homodimers to regulate downstream gene expression. RORA has been implicated in various physiological processes, including embryonic development, cell differentiation, immune regulation, circadian rhythm, and the metabolism of lipids, steroids, xenobiotics, and glucose.^[^ ^44^, ^45^ ^]^ Previous studies have shown that RORA has both proinflammatory and anti‐inflammatory functions depending on the cellular context. For example, Rora deficiency in mast cells and macrophages leads to increased production of TNF‐α and IL‐6 upon activation,^[^ ^46^ ^]^ whereas ectopic expression of RORα1 in human primary smooth muscle cells suppresses TNF‐α‐induced expression of IL‐6, IL‐8, and COX‐2 through upregulation of the inhibitory protein IκBα.^[^ ^47^ ^]^ Proinflammatory roles of RORA have also been reported in various cell types.^[^ ^48^, ^49^ ^]^

In our study, increased histone lactylation at the Rorα promoter was associated with increased Rorα expression, which in turn upregulated the transcription of proinflammatory genes, including *il1, il6, il17a/f1, cebpb* and *cxcl8a*. These changes promote neutrophil activation, migration, and the release of inflammatory mediators, ultimately contributing to systemic immune activation and immune‐mediated tissue damage. To further support the clinical relevance of our findings, we conducted a preliminary sleep deprivation study in human volunteers. After 24 h of continuous wakefulness, peripheral blood analysis revealed a significant increase in both the percentage and absolute count of circulating neutrophils, as well as elevated serum lactate levels (Figure S6A–D, Supporting Information). These physiological changes mirror the acute neutrophilic response and metabolic alterations observed in our murine models, suggesting that even short‐term sleep deprivation in humans can induce comparable immunometabolic effects. Additionally, to assess the conservation of the identified mechanism in humans, we performed in vitro validation using commercially sourced primary human peripheral blood neutrophils. Lactate supplementation significantly increased H3K18 lactylation levels in cultured human neutrophils (Figure S6E,F, Supporting Information), accompanied by the upregulation of RORα and its downstream transcription factors (CEBPA, CEBPB, CEBPE, GFI1) (Figure S6G–K, Supporting Information). Furthermore, this metabolic stimulation increased the expression of key inflammatory cytokines, including IL‐1β, IL‐6, and IL‐8 (Figure S6L–N, Supporting Information). These findings suggest that the lactate–H3K18 lactylation–RORα axis is at least partially conserved in human neutrophils and may underlie the similar inflammatory priming observed during sleep deprivation. Although these results were obtained under simplified in vitro conditions, they nonetheless support the translational relevance of our murine and zebrafish models (Figure S6A–N, Supporting Information). These findings expand our understanding of how lactate metabolism can influence immune responses at the epigenetic level and offer a novel perspective on the metabolic regulation of immune function.

Furthermore, our study contributes to the growing body of literature on the role of glycolysis in neutrophil activation and immune responses. Previous studies have shown that glycolysis plays a central role in supporting neutrophil function during inflammation by increasing neutrophil phagocytic capacity, ROS production, and NET formation.^[^ ^41^, ^50^ ^]^ We confirmed these findings, showing that sleep deprivation induces glycolytic reprogramming in neutrophils, which contributes to their activation and subsequent immune dysregulation. The involvement of lactate in this process is particularly noteworthy, as lactate has been shown to regulate immune cell function beyond its role in energy metabolism. Lactate‐induced histone lactylation serves as an epigenetic switch that increases the expression of genes critical for neutrophil activation and immune responses.

In addition to the metabolic alterations observed in neutrophils, we also noted a significant systemic impact of sleep deprivation on immune function. Notably, neutrophil counts peaked at 24 h of sleep deprivation but declined slightly at 48 h. This biphasic pattern may reflect a dynamic regulatory response of the immune system to sustained sleep loss. One plausible explanation is that acute sleep deprivation acts as a physiological challenge sufficient to trigger transient inflammatory activation, promoting the mobilization of neutrophils from the bone marrow and marginated pools into the circulation. Over time, compensatory mechanisms—such as neuroendocrine adaptation, negative feedback regulation of inflammatory pathways, or enhanced neutrophil clearance—may contribute to the subsequent reduction in the number of circulating neutrophils. Alternatively, prolonged wakefulness may initiate immunoregulatory programs or lead to hematopoietic fatigue, thereby attenuating the neutrophil response. Future studies employing longitudinal tracking of bone marrow output and tissue‐resident neutrophils will be valuable to further elucidate these mechanisms.

Our results indicate that sleep deprivation induces systemic immune dysregulation characterized by a cytokine storm, which is largely driven by neutrophil activation. Sleep loss disrupts the neuroimmune balance, with acute deprivation amplifying cytokine release and chronic loss driving low‐grade inflammation linked to cardiometabolic and neurodegenerative diseases.^[^ ^13^ ^]^ This phenomenon, known to be associated with various inflammatory disorders, may contribute to the pathological consequences of sleep deprivation, including immune‐mediated tissue damage and increased mortality. This inflammatory cascade appears to be orchestrated by the interplay between glycolytic reprogramming, lactate accumulation, and histone lactylation, which collectively promote immune cell activation and subsequent inflammatory responses. Although both 2‐DG and OXA significantly attenuated the neutrophilic response to sleep deprivation, they did not completely reverse the immune alterations observed. This finding suggests that, beyond glycolytic reprogramming, other mechanisms may contribute to SD‐induced neutrophil activation. Emerging evidence indicates that neuroendocrine factors, oxidative stress, and circadian disruption can all modulate immune cell dynamics and may collectively shape the inflammatory response during sleep loss.^[^ ^3^ ^,^ ^51^ ^]^ These possibilities warrant further investigation and may explain why the inhibition of glycolysis alone is insufficient to fully suppress neutrophil recruitment following SD.

While our study provides important insights into the interplay between sleep deprivation, innate immunity, and metabolic regulation, several limitations warrant discussion. The CPW paradigm employed in our study was chosen for its unique ability to maintain a high level of wakefulness (>95%) over extended periods, effectively minimizing microsleep episodes that commonly occur in traditional paradigms such as gentle handling, rotating wheels, or multiple platform setups. Unlike these labor‐intensive and often inconsistent methods, CPW achieves robust sleep suppression with minimal human interference. We acknowledge that sleep deprivation itself constitutes a physiological stressor. In our study, although serum corticosterone levels tended to slightly increase following CPW exposure, no statistically significant differences were observed compared with those in control animals (Figure S1A,B, Supporting Information). These findings indicate that the CPW‐induced endocrine response was modest and within the range of normal physiological variability. We therefore consider the CPW method to introduce a mild but well‐controlled physiological perturbation—sufficient to elicit the early immune alterations associated with acute sleep loss—while avoiding the confounding severity observed in classic restraint‐induced stress models. Moreover, no significant changes in food intake were detected during the first 72 h (Figure S1D,F, Supporting Information), further supporting the minimal metabolic disruption induced by this paradigm.

Importantly, our aim was not to artificially isolate sleep deprivation from all stress‐related effects—an inherently challenging distinction—but to investigate how early immune dynamics, particularly neutrophil activation and cytokine expression, respond to sustained wakefulness under controlled experimental conditions. Our findings highlight a specific metabolic‒epigenetic axis (lactate–H3K18la–RORα) that may underlie this response, and this mechanism has been consistently observed across species. Overall, we recognize that while some degree of physiological stress is unavoidable in any SD model, the CPW paradigm allows for efficient, reproducible induction of sleep loss with acceptable and controlled systemic effects. Future refinements to this model and human validation will further strengthen its translational utility.

In summary, our study provides new insights into the role of lactate metabolism and histone lactylation in regulating immune responses during sleep deprivation. We propose that sleep deprivation‐induced metabolic reprogramming, specifically glycolysis and lactylation modifications, plays a critical role in driving neutrophil activation and systemic inflammation. These findings open new avenues for exploring therapeutic strategies targeting lactate metabolism and epigenetic modifications to modulate immune responses in individuals suffering from sleep deprivation. Future research will further elucidate the mechanisms underlying lactate metabolism and immune regulation, with potential implications for the treatment of immune‐related diseases and chronic sleep loss.

## 4 Conclusion

In conclusion, our findings delineate a conserved metabolic‒epigenetic mechanism by which acute sleep deprivation drives systemic neutrophilic inflammation. We demonstrated that elevated glycolysis and lactate accumulation promote histone H3K18 lactylation, which in turn transcriptionally activates Rorα, a key regulator of neutrophil function. Disruption of this axis via genetic or pharmacological interventions effectively attenuates neutrophil overactivation and downstream inflammation. Importantly, this lactate‐H3K18la‐Rorα pathway is conserved across mice, zebrafish, and pigs, underscoring its translational relevance. These results highlight the critical interplay between sleep, metabolism, and immunity and provide a mechanistic framework for targeting sleep loss–induced inflammatory disorders.

## 5 Experimental Section

### Experimental Animals

The animal experiments were approved by the Ethics Committee of Anhui Agricultural University (No. KJLLXM2025156). Seven‐ to eight‐week‐old male C57BL/6J mice with similar initial body weights were randomly assigned to each experimental group. The mice were maintained in a vivarium with a 12‐h light/dark cycle at 22–24 °C. Food and water were available ad libitum. The animals were randomly assigned to each treatment group. All personnel were blinded during the animal experiments, and no samples or animals were intentionally excluded from the analyses. For zebrafish, embryos were collected from wild‐type (WT) zebrafish, the transgenic Tg(mpx:EGFP) line, and the *rora^−/−^* mutant model following natural mating at 28.5 °C. The embryos were then placed in E3 embryo medium and handled according to the zebrafish breeding guidelines outlined in the Zebrafish Book. Wild‐type (WT) and Tg(*mpx:EGFP*) transgenic zebrafish embryos were cultured with Hank's solution at a constant temperature of 28.5 °C, pH of 6.5–7.5, under 14 h/10 h light and dark (LD) (light at 8:30 a.m., dark at 10:30 p.m., ZT0 = light). N‐phenylthiourea (PTU, Sigma, USA) was used to prevent pigment formation in larvae. The sex of the juvenile fish did not differ at this time, so there was no sex difference in the experimental animals in this study. Sixty‐day‐old male pigs with similar initial body weights were randomly assigned to each experimental group. Pigs were housed in a well‐ventilated facility with a natural light/dark cycle and maintained at a comfortable ambient temperature (22–24 °C). Food and water were provided ad libitum throughout the study. The animals were randomly assigned to each treatment group. All personnel conducting the experiments were blinded to the group allocations, and no samples or animals were intentionally excluded from the analyses. Efforts were made to minimize animal stress and suffering and reduce the number of animals used in compliance with ethical guidelines.

### Sleep Deprivation

On the basis of the protocol from Sang et al.^[^ ^14^ ^]^ we developed customized acrylic cylinders for sleep deprivation (SD) experiments in mice. The mice were divided into two groups: the SD group and the Con group. For the SD group, the mice were placed in cylinders containing an 8 mm deep layer of water, preventing them from finding a dry spot to rest, thereby inducing sleep deprivation. The water was changed every 24 h to maintain cleanliness. The mice in the control (Con) group were housed under identical conditions (same cage size, temperature, and light cycle) with free access to food and water. For zebrafish larval sleep deprivation, we followed the protocol of Leung et al.^[^ ^29^ ^]^ Four‐day post fertilization (dpf), larvae were placed in 15 mL of system water in 15‐mL conical centrifuge tubes (Falcon, 352 097). A 2.5 mL air bubble was created by removing sufficient liquid to cover the 2.5 mL graduating tube. The tube was placed on a 360° rotating platform set to 0.5 Hz, causing the larvae to be disturbed by the air bubble. The larvae instinctively moved to the tip of the tube, avoiding turbulence, but were forced to return to the center when they drifted away during sleep. For sleep deprivation in pigs, a modified protocol based on video surveillance and manual intervention was used. Pigs were housed individually in pens equipped with continuous 24‐h infrared video monitoring. Sleep and wake states were assessed on the basis of movement, postural changes, and behavioral cues. When pigs exhibited sleep‐like behaviors, such as lying down and reduced movement, trained personnel applied gentle but persistent stimuli, including auditory cues (clapping or calling) and mild tactile stimulation (nudging with a soft object), to prevent sleep without causing undue stress. Owing to their larger body size, pigs were subjected to a longer period of sleep deprivation (96 h) to ensure the effectiveness of the intervention. The control pigs were housed under identical conditions but without external interventions. For sleep deprivation in humans, six healthy adult volunteers were recruited and subjected to a controlled sleep deprivation protocol. Peripheral blood samples were collected twice: once after a night of habitual sleep (control condition), and again following 24 h of continuous wakefulness (sleep deprivation condition). Participants remained awake under monitored laboratory conditions and were allowed to engage in low‐intensity activities (e.g., reading, conversation) but were prohibited from napping or lying down. Compliance was ensured through direct supervision. All participants provided written informed consent prior to the study. This protocol was reviewed and approved by the Ethics Committee of Anhui Agricultural University (No. KJLLXM2025156).

### EEG/EMG‐Based Sleep/Wake Analysis

For electrode implantation, C57BL/6J male and female mice (7–8 weeks old) were randomly assigned to the experimental group. They were housed in a constant temperature (22–24 °C) chamber with a 12‐h light/dark cycle and had free access to food and water. The mice were anesthetized with isoflurane and secured in a stereotactic device for surgery. The skull was exposed, and two stainless steel microscrews were implanted at predefined coordinates for EEG recordings. Two EMG microwires were inserted into the neck muscles. The connector was fixed with dental cement, and the mice were allowed to recover for 48 h. EEG/EMG data were recorded continuously for 24 h at a 1000 Hz sampling frequency and 50 Hz filter frequency. Sleep stages were analyzed via machine learning algorithms, Z scores, and pretrained convolutional neural networks (CNNs). Statistical analysis was performed via customized MATLAB scripts.

### Restraint Stress Model

To induce restraint stress, the mice were subjected to intermittent restraint sessions in 50 mL polypropylene conical tubes with ventilation caps. Stress protocols were initiated between 8:00 and 9:00 AM, consisting of 20‐min restraint intervals alternating with 40‐min rest periods over a 24‐h duration. This cycle (20 min stress → 40 min rest) was repeated to ensure controlled stress exposure while preventing prolonged immobility complications.

### Corticosterone Measurement

Blood was collected from the mice (9–11 AM to minimize circadian effects). The serum was separated via centrifugation (3000×g, 15 min, 4 °C) and stored at −80 °C. Corticosterone levels were quantified via a species‐specific ELISA kit (Abcam #ab108821). The samples (1:10 dilution) and standards were incubated (37 °C, 1 h), washed, and developed with TMB. The absorbance (450 nm) was measured, and concentrations (ng/mL) were calculated against a standard curve.

### Cortisol Measurement

Saliva was collected from pigs via synthetic swabs rubbed gently against the buccal mucosa for 30 s. Samples were centrifuged (3000 × *g*, 10 min, 4 °C) to remove debris and stored at −80 °C. Cortisol levels were quantified via a porcine‐validated ELISA kit (Salimetrics #1–3002). Undiluted samples and standards were incubated (25 °C, 1 h), washed, and reacted with TMB. The absorbance (450 nm) was measured, and the concentrations (ng mL^−1^) were calculated against a standard curve.

### Feed Intake Measurement

Feed consumption was recorded daily via a precision scale. Mice: Preweighed chow was provided to individually housed animals; residual feed was weighed at 24 h intervals. Pigs: Individually housed pigs received weighed feed portions; leftovers were collected, dried (60 °C, 24 h), and reweighed.

### Data Collection

Here, we retrieved single‐cell RNA sequencing data (GSE213496, available at https://www.ncbi.nlm.nih.gov/geo/query/acc.cgiacc = GSE213496) from the Gene Expression Omnibus (GEO) database and obtained glycolysis‐related and lactylation‐related gene sets from the Gene Set Enrichment Analysis (GSEA) database (https://www.gsea‐msigdb.org/gsea/index.jsp).

### Single‐Cell RNA Sequencing Data Processing

Single‐cell sequencing data were obtained from the GEO database, followed by data normalization and dimensionality reduction via Seurat V4. Glycolysis scores for each cell were calculated via the AddModuleScore function. The following cell types were annotated on the basis of canonical marker genes: B cells (Cd19, Ms4a1, Cd79a, Mzb1, and Cd2), neutrophils (S100a8, S100a9, Csf3r, and Cxcr2), T&NK cells (Cd2, Cd3d, Cd3e, Cd8a, and Nkg7), monocytes/macrophages (Cd68), and erythrocytes (Hbb‐bt and Alas2). To investigate the effects of sleep deprivation on neutrophils, differential gene expression analysis was performed between the 24‐h sleep‐deprived and control groups via the Wilcoxon rank‐sum test, with an adjusted p value < 0.05 as the significance threshold. Subsequently, KEGG pathway enrichment analysis was conducted on the DEGs via the clusterProfiler 4.6.2 package, with the significance level set at *p* < 0.05.

### Pseudotime Analysis

Cell trajectories were constructed via the Monocle 2.26.0 R package. Differentially expressed genes were used to construct trajectory plots. Through dimensionality reduction analysis, cells were ordered on the basis of transcriptomic similarity to obtain a pseudotime axis, displaying the developmental trajectories at 0 h, 24 h, and 48 h of sleep deprivation.

### In Vivo Treatments

To promote glycolysis and lactylation, sodium l‐lactate or d‐lactate was administered. In mice, sodium l‐lactate or d‐lactate (50.5 mg kg^−1^ body weight, dissolved in PBS) was administered via intraperitoneal injection, and tissue samples were collected 4 h postinjection. In zebrafish, sodium l‐lactate or d‐lactate (50 × 10^−3^ m, diluted in Hank's solution) was added to the tank water for 4 h prior to sample collection or imaging following tail fin injury. An additional d‐lactate (d‐Lac) control group, representing the inactive stereoisomer, was included to assess the specificity of lactate‐induced immunomodulatory effects and to exclude nonspecific metabolic stress as a confounding factor. To inhibit glycolysis and reduce lactate production, 2‐deoxy‐glucose (2‐DG) and sodium oxamate (OXA) were used. The mice were intraperitoneally injected with 2‐deoxy‐d‐glucose (2‐DG, 50 × 10^−3^ m in PBS) or oxamate (OXA, 750 mg kg^−1^ body weight in PBS) 3 h before the onset of sleep deprivation and again 24 h after SD initiation. Samples were collected after 48 h of continuous sleep deprivation. Zebrafish larvae were subjected to sleep deprivation in drug‐containing water for 24 h. The treatment solutions contained either 2‐deoxy‐d‐glucose (2‐DG, 30 × 10^−3^ m in Hank's buffer) or oxamate (OXA, 10 × 10^−3^ m in Hank's buffer). Samples were collected immediately after the 24‐h sleep deprivation period. Both 2‐DG and OXA interventions were aimed at downregulating lactylation modifications by targeting glycolysis and lactate production.

### H&E Staining

Livers, spleens, kidneys and small intestines were fixed with 4% paraformaldehyde (PFA) overnight and embedded in paraffin. Sections were cut at a thickness of 10 µm. The experiment followed the standard H&E staining protocol. Briefly, after dewaxing and rehydrating, the nuclei were stained with hematoxylin (Sigma, 03971) for 3 min and differentiated with acid alcohol for 30 s. The cytoplasm was stained with eosin (Sigma, 318906) for 10 s. The slices were dehydrated and sealed with neutral resin. The slices were scanned via a VS120 Virtual Slide Microscope (Olympus Life Sciences).

### Immunofluorescence Staining

Neutrophils were isolated from mouse and pig blood, fixed in 4% paraformaldehyde (PFA) for 15 min at room temperature, and permeabilized with 0.1% Triton X‐100 for 10 min. After being blocked with 5% bovine serum albumin (BSA) for 1 h at room temperature, the samples were incubated with primary antibodies against pan‐Kla or H3K18la overnight at 4 °C. The following day, the cells were washed with PBS and incubated with fluorescently labeled secondary antibodies for 1 h at room temperature. Nuclei were counterstained with DAPI for 10 min. Images were captured via a laser scanning confocal microscope, and the fluorescence intensity was analyzed via ImageJ software. All the experiments were performed in triplicate to ensure consistency.

### Immunofluorescence of Neutrophils in the Liver and Spleen

Liver and spleen tissues from mice and pigs were harvested immediately after euthanasia and fixed in 4% paraformaldehyde (PFA) at 4 °C overnight. Fixed tissues were embedded in paraffin, sectioned into 4‐µm‐thick slices, and subsequently deparaffinized in xylene followed by rehydration through a graded ethanol series (100%, 95%, 70%) and distilled water. Antigen retrieval was performed by heating the slides in citrate buffer (pH 6.0) for 15 min, followed by cooling to room temperature. The sections were then permeabilized with 0.1% Triton X‐100 in PBS for 10 min and blocked with 5% bovine serum albumin (BSA) in PBS for 1 h at room temperature to minimize nonspecific binding. The slides were incubated overnight at 4 °C with primary antibodies against Ly6G (mouse neutrophils) and MPO (myeloperoxidase); for pig samples, a species‐specific neutrophil marker antibody was used. After washing, fluorescent secondary antibodies (e.g., Alexa Fluor 488 or 594) were applied for 1 h at room temperature in the dark, followed by nuclear staining with 4′,6‐diamidino‐2‐phenylindole (DAPI) for 5 min. The slides were mounted with antifade mounting medium, and images were captured via a laser confocal microscope. Fluorescent signals for neutrophils (Ly6G, MPO) and nuclei (DAPI) were quantified via ImageJ software. Appropriate negative controls (e.g., omission of primary antibodies) were included, and species‐specific antibody compatibility was validated in preliminary experiments.

### CUT&Tag Sequence

CUT&Tag was conducted according to the Hyperactive Universal CUT&Tag Assay Kit instructions (Vazyme Biotech, TD903). Neutrophils from both control and sleep‐deprived mice were incubated with ConA beads, followed by overnight incubation with H3K18la antibodies. Secondary antibodies and pA/G‐Tnp transposons were added to activate translocase for DNA extraction. The DNA library was prepared via the TruePreP Index Kit V2 and sequenced on the Illumina NovaSeq platform.

### Neutrophil Isolation

Blood was collected from anesthetized mice, pigs, human and neutrophils were isolated via a neutrophil isolation kit (Solarbio, P9201). Fresh anticoagulated blood was taken, and 4 mL of reagent A and 2 mL of reagent C were sequentially added to a centrifuge tube, ensuring that a clear density interface formed. At room temperature, centrifugation was performed at 500–1000 × *g* for 20–30 min to separate the neutrophil layer. The neutrophils were precisely aspirated via a pipette and transferred to a clean 15 mL centrifuge tube. The samples were then washed with PBS or cell washing solution, followed by centrifugation at 250 × *g* for 10 min to remove contaminating red blood cells. The cells were resuspended in PBS or washing solution and centrifuged again at 250 × *g* for 10 min. This washing and centrifugation process was repeated to ensure purity. Finally, the supernatant was discarded, the cells were resuspended for cellular immunofluorescence, and RNA and proteins were extracted for WB experiments. Flushed BM cells were resuspended in 200 µL of MACS buffer (2 mM EDTA, 0.5% bovine serum albumin in PBS), and BM neutrophils were isolated according to the protocol of the “Neutrophil Isolation Kit, mouse” by Miltenyi. The yields were ≈4 × 106 cells in total (for 4 bones of two legs in mice).

### l‐Lactate Measurement

L‐lactate levels in various samples, including BW, blood neutrophil, heart, liver, spleen, lung, and muscle samples, were measured via a fluorescence‐based l‐lactate assay kit (Cayman, 700510) following the manufacturer's instructions. BW was extracted from four bones (femurs and tibias) per mouse, washed with 1 mL of PBS solution, and processed to measure l‐lactate. Peripheral blood mononuclear cells (PBMCs) were isolated via density gradient centrifugation, and neutrophils were separated for l‐lactate detection via the same fluorescence‐based assay. Tissue samples from the heart, liver, spleen, lung, and muscle were collected, weighed, and homogenized in ice‐cold PBS via a tissue homogenizer. The homogenates were centrifuged at 12000 × *g* for 10 min at 4 °C to obtain the supernatants, which were analyzed via the l‐lactate assay. The fluorescence intensities were measured via a microplate reader (excitation/emission: 530/590 nm), and the L‐lactate concentrations were calculated from the standard curve provided with the kit. All measurements were performed in triplicate to ensure accuracy and reproducibility, allowing for a comprehensive assessment of L‐lactate levels across different physiological compartments in response to experimental conditions.

### Zebrafish Behavior Test

Due to technical limitations, real‐time sleep behavior could not be recorded during the sleep deprivation process. Instead, postdeprivation behavioral monitoring was conducted to evaluate alterations in sleep architecture, as referenced in previous studies. Louis C et al. The sleep behavior of zebrafish larvae was assessed via the ZebraLab 3.10 video tracking system (ViewPoint Life Sciences, France). Zebrafish larvae were raised under a standard 14 h:10 h light‒dark (LD) cycle until 5 dpf. On the day of the assay, morphologically normal larvae were transferred individually into the wells of a 48‐well plate, with one larva per well in equal volumes of Hanks' solution. The plate was carefully placed into the behavior recording chamber, and the lid was closed to prevent external light interference. Two groups were included in the experiment: a control group (Con) and a group subjected to 24‐h sleep deprivation (SD24h). The experiment was set up in “Quantization‐Live” mode, with detection zones defined for each well. The activity algorithms were set to “Activity” and “Activity Sum,” with a high activity threshold of 25 and an immobility threshold of 4. The behavioral data were continuously recorded for 24 h, with a sampling frequency of one data point per min. Lighting conditions were programmed to mimic the standard LD cycle (lights on at 08:30 with 50% brightness, lights off at 22:30). The raw movement data were exported and analyzed via Microsoft Excel. Sleep was defined as any period of continuous inactivity lasting at least 1 min. The amount of sleep was calculated in 10‐min bins, and total daytime and nighttime sleep durations were quantified for each larva.

### Caudal Fin Injury and Imaging

Tg(mpx:EGFP) larvae (5 dpf) were anesthetized with 0.1 g mL^−1^ MS‐222 solution (Sigma, E10521). The tail fins were surgically damaged via blades under a stereomicroscope. Three h after injury, the migration of neutrophils in the caudal fin was observed via fluorescence microscopy, and the number of neutrophils was analyzed via ImageJ software. A total of 15 zebrafish were used in each group.

### RNA Extraction and qPCR

Total RNA was extracted from zebrafish larvae, murine neutrophil cells, porcine neutrophils and other tissues via a SPARK easy Tissue/Cell RNA Rapid Extraction Kit (SPARKjade, AC0202, Shandong Sparkjade Biotechnology Co., Ltd.), and reverse transcription was performed via a SPARKScript RT plus Kit (SPARKjade, AG0304). Real‐time quantitative PCR (Q‐PCR) was conducted via a SYBR Green kit (Takara, Japan). Approximately 40 cycles at 95 °C for 10 seconds and 60 °C for 30 seconds were conducted to amplify the related genes. The Q‒PCR experiments were repeated with three individual biological samples. The data were normalized to the expression level of the housekeeping gene β‐actin, and the relative expression levels were calculated via the 2^(‐ΔΔCt)^ method.

### Western Blotting

The lactation level of histone protein after treatment with different drugs was analyzed. After drug treatment, zebrafish larvae and cells from each group were collected, centrifuged, and lysed in RIPA buffer (Servicebio, China) for Western blotting. The collected samples were boiled for 5 min and electrophoresed on a 12.5% SDS‒PAGE gel. The nitrocellulose membrane was blocked with 5% milk and incubated overnight with antibodies against H3K18la (1:1000, PTM‐1427, PTMBIO), actin (1:1000, HuaBIO), pan‐Kla (1:1000, PTM‐1401, PTMBIO) or Tubulin (1:1000, HuaBIO). After washing, the membranes were incubated with an HRP‐labeled secondary antibody (Sangon, D111042‐0100) for 2 h at room temperature and then photographed with chemiluminescent solution.

### Plasmid Construction

The full‐length zebrafish *cebpb* coding sequence was PCR‐amplified via gene‐specific primers with engineered restriction sites and cloned and inserted into the pcDNA3.1(+) vector (Invitrogen) between the BamHI and XbaI restriction sites. The resulting construct, pcDNA3.1‐*cebpb*, was verified by restriction digestion and Sanger sequencing. The expression cassette is driven by the CMV promoter and contains a neomycin resistance gene (Neo) and polyadenylation (PolyA) signal. The construct was used for *cebpb* overexpression experiments in zebrafish embryos via microinjection.

### Human Neutrophil Culture

Human peripheral blood mononuclear cells from healthy donors were purchased from Milestone Biotechnologies (Beijing, China). Informed consent was obtained from all donors before specimen collection, following the principles of the Declaration of Helsinki. The cells were cultured in RPMI‐1640 medium supplemented with 10% fetal bovine serum (FBS) and 1% penicillin‒streptomycin at 37 °C in a humidified 5% CO₂ incubator. To simulate a lactate‐enriched environment and assess the effects of metabolic modulation, the cells were treated with l‐lactate (30 × 10^−3^ m), d‐lactate (30 × 10^−3^ m, an inactive stereoisomer control), 2‐deoxy‐d‐glucose (2‐DG, 50 × 10^−3^ m), or oxamate (OXA, 30 × 10^−3^ m) for 4 h. Following treatment, the neutrophils were collected for RNA/protein extraction and gene expression analysis.

### Statistical Analysis

The data are expressed as the means ± SD. Comparisons between two groups were made with an unpaired t test, and comparisons between more than two groups were made with one‐way ANOVA followed by the Bonferroni post hoc correction. The Kruskal‒Wallis test was used to compare glycolysis and lactylation scores across different cell types or clusters. All analyses were performed with GraphPad software (GraphPad Software 8.4.3). Data were considered statistically significant if *P* < 0.05 (****P < 0.0001, ***P < 0.001, **P < 0.01, *P < 0.05).

## Conflict of Interest

The authors declare no conflict of interest.

## Author Contributions

R.Z., K.L., and X.H. contributed equally to this work. D.L. Ren designed and supervised the study. R.Z., K.Y.L., X.Z.H., Y.X.G., X.S.X., Y.B., H.Y.Z., Y.L.W., C.J.W., Z.W.X., S.H.F., Z.J.Y., S.R.Z., C.L., and P.C. conducted the experiments. D.L.R. and R.Z. drafted and revised the manuscript.

## Supporting information



Supporting Information

## Data Availability

The data that support the findings of this study are available from the corresponding author upon reasonable request.;
