# Correction to “Sleep Deprivation Activates a Conserved Lactate‐H3K18la‐RORα Axis Driving Neutrophilic Inflammation Across Species”

**DOI:** 10.1002/advs.202521324

**Published:** 2025-11-06

Zhou R, Li K, Hu X, Fan S, Gao Y, Xue X, Bu Y, Zhang H, Wang Y, Wei C, Zhang S, Xie Z, Liu C, Chen P, Yin Z, Ren D.

DOI: 10.1002/advs.202504028

Adv. Sci. (Weinh). 2025 Oct;12(38):e04028. Epub 2025 Jul 21. PMID: 40686333; PMCID: PMC12520482.

In the published version of our article, a typesetting error occurred, resulting in an incorrect image usage in Figure S4I (Supporting Information) (the rest of the article's figures are accurate and unaffected). The details are as follows: The image designated for the “L‐Lac (L‐lactate) group” in Figure S4I (Supporting Information) Representative images of neutrophil migration in the zebrafish caudal fin injury model″—was mistakenly selected during the typesetting process.

To rectify this error, we have prepared the correct image specifically for the L‐Lac group in Figure S4I (Supporting Information), which accurately depicts the neutrophil migration characteristics corresponding to the L‐lactate treatment condition. It is crucial to emphasize that this correction only involves the image of the L‐Lac group in Figure S4I (Supporting Information); it does not alter the article's original research findings, compromise the reliability of the underlying experimental data, or impact the integrity of the statistical analyses presented.


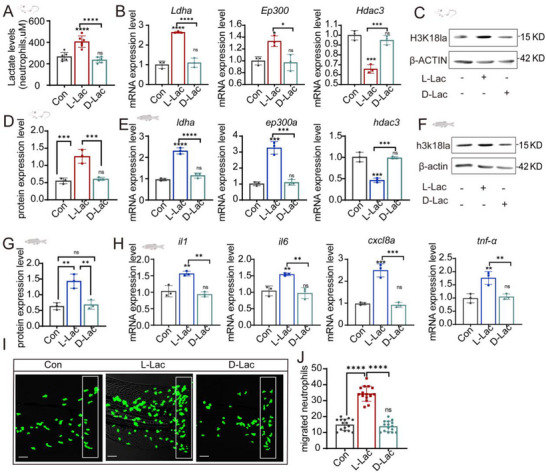


We apologize for this error.

