# Supplementary material for: Sleep Deprivation Activates a Conserved Lactate‐H3K18la‐RORα Axis Driving Neutrophilic Inflammation Across Species
Source: Adv Sci (Weinh). 2025 Jul 21;12(38):e04028. doi: 10.1002/advs.202504028 (PMC12520482; doi:10.1002/advs.202504028)
Supplement: Supplementary file 1 — Supporting Information [file ADVS-12-e04028-s001.docx]

Supporting Information

Sleep Deprivation Activates a Conserved Lactate-H3K18la-RORα Axis Driving Neutrophilic Inflammation across Species

Ren Zhou^#^, Keyun Li^#^, Xiezong Hu^#^, Shuhao Fan^*^, Yuxuan Gao,Xiaoshu Xue,Yu Bu, Haoyi Zhang, Yili Wang, Chunjiao Wei, Shangrong Zhang, Zhongwen Xie, Chao Liu, Peng Chen, Zongjun Yin^*^, Dalong Ren^*^

R Zhou, KY Li, XZ Hu, YX Gao, HY Zhang, XS Xue, B Yu, YL Wang, CJ Wei, ZW Xie, SH Fan, ZJ Yin, DL Ren, College of Animal Science and Technology, Anhui Agricultural University, Hefei 230036, China.

*SR Zhang, Anhui Province Key Laboratory of Embryo Development and Reproductive Regulation, Fuyang Normal University, Fuyang 236037, China*

*C Liu, School of Life Sciences, Suzhou Medical College of Soochow University, Suzhou 215123, China*

*P Chen, Institute of Brain Science, The First Affiliated Hospital of Anhui Medical University, Hefei 230022, China.*

E-mail: *Corresponding author, Dalong Ren, rendl@ustc.edu.cn


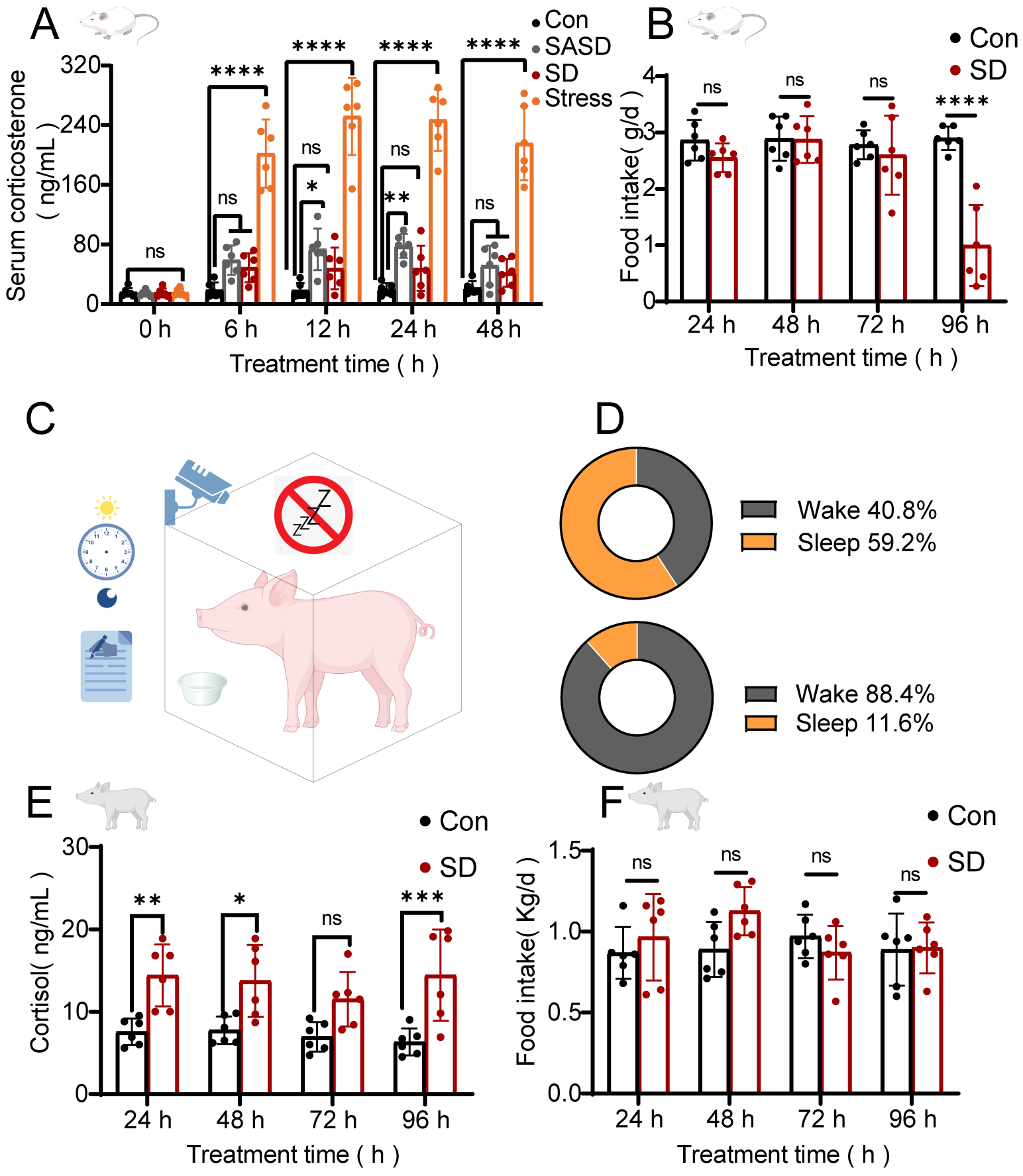


**Supplementary Figure 1. Assessment of stress levels and food intake in sleep-deprived mice and pigs. A) Serum corticosterone levels in mice subjected to different treatments: control (Con), stand-alone SD (SASD), sleep deprivation (SD), and restraint stress (Stress) at indicated time points (0, 6, 12, 24 h and 48 h). B) Daily food intake in mice under Con or SD conditions over 24–96 h. A significant reduction in intake was observed only at 96 h. C) Schematic of the sleep deprivation setup for pigs, involving continuous human monitoring and behavioral intervention to prevent sleep. D) Pie charts showing the sleep/wake distribution in pigs under Con and SD conditions across a 24-h cycle. SD significantly reduced total sleep time. E) Cortisol levels in pigs under Con or SD conditions at 24, 48, 72, and 96 h. F) Daily food intake in pigs under Con or SD conditions; no significant differences were observed. The data are shown as the means ± SD. The data were analyzed via unpaired t tests or one-way ANOVA with Tukey’s post hoc test, with significance levels denoted as follows: ns, p > 0.05; *, p < 0.05; **, p < 0.01; ***, p<0.001; and ****, p<0.0001.**

**
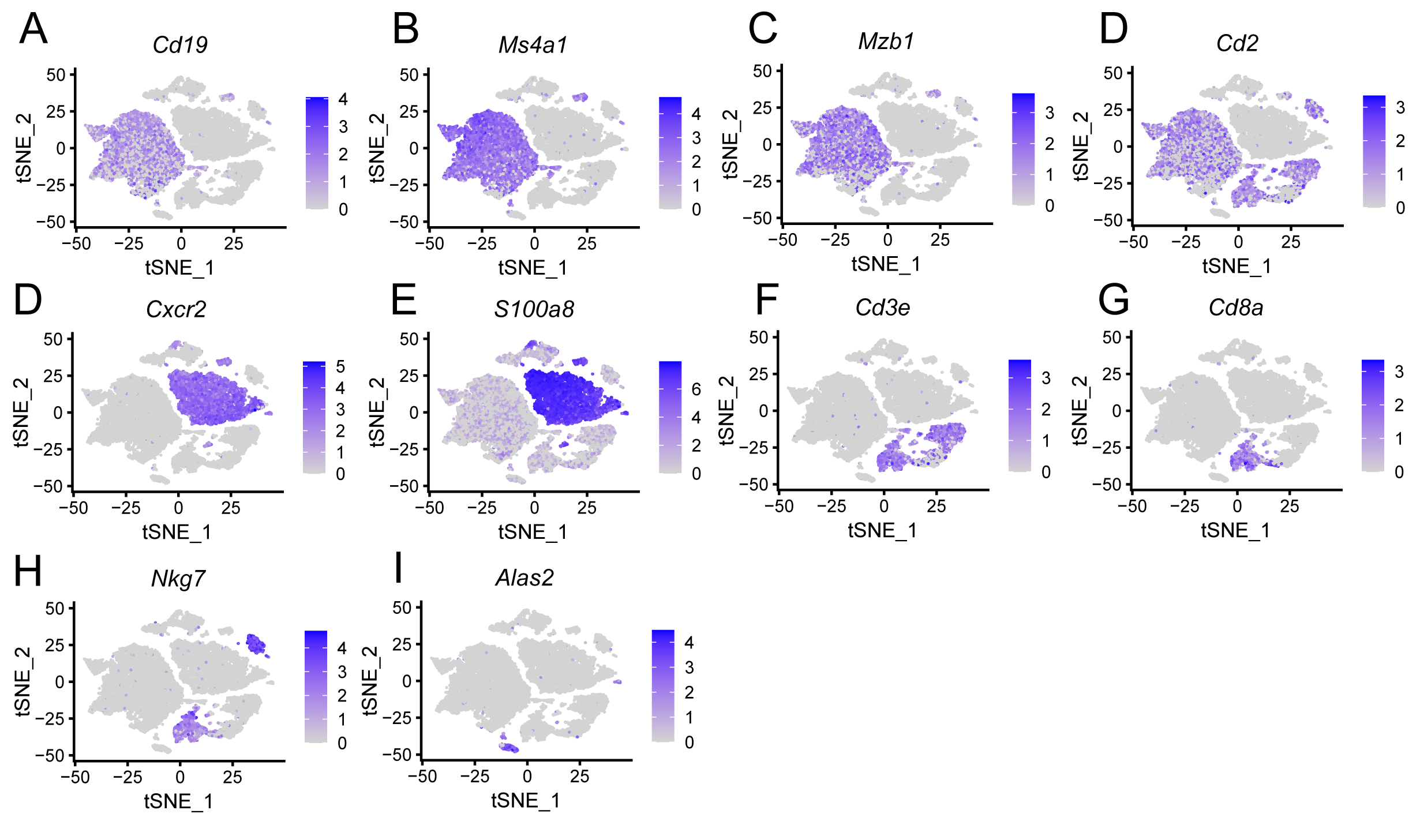
**

**Supplementary Figure 2. Feature plots of cell type-specific marker genes in single-cell RNA sequencing data. A) *Cd19* (B cells), B) *Ms4a1* (B cells), C) *Mzb1* (B cells), D) *Cd2* (T cells), E) *Cxcr2* (neutrophils), F) *S100a8* (neutrophils), G) *Cd3e* (T cells), H) *Cd8a* (CD8+ T cells), I) *Nkg7* (cytotoxic cells), J) *Alas2* (erythroid cells).**

**
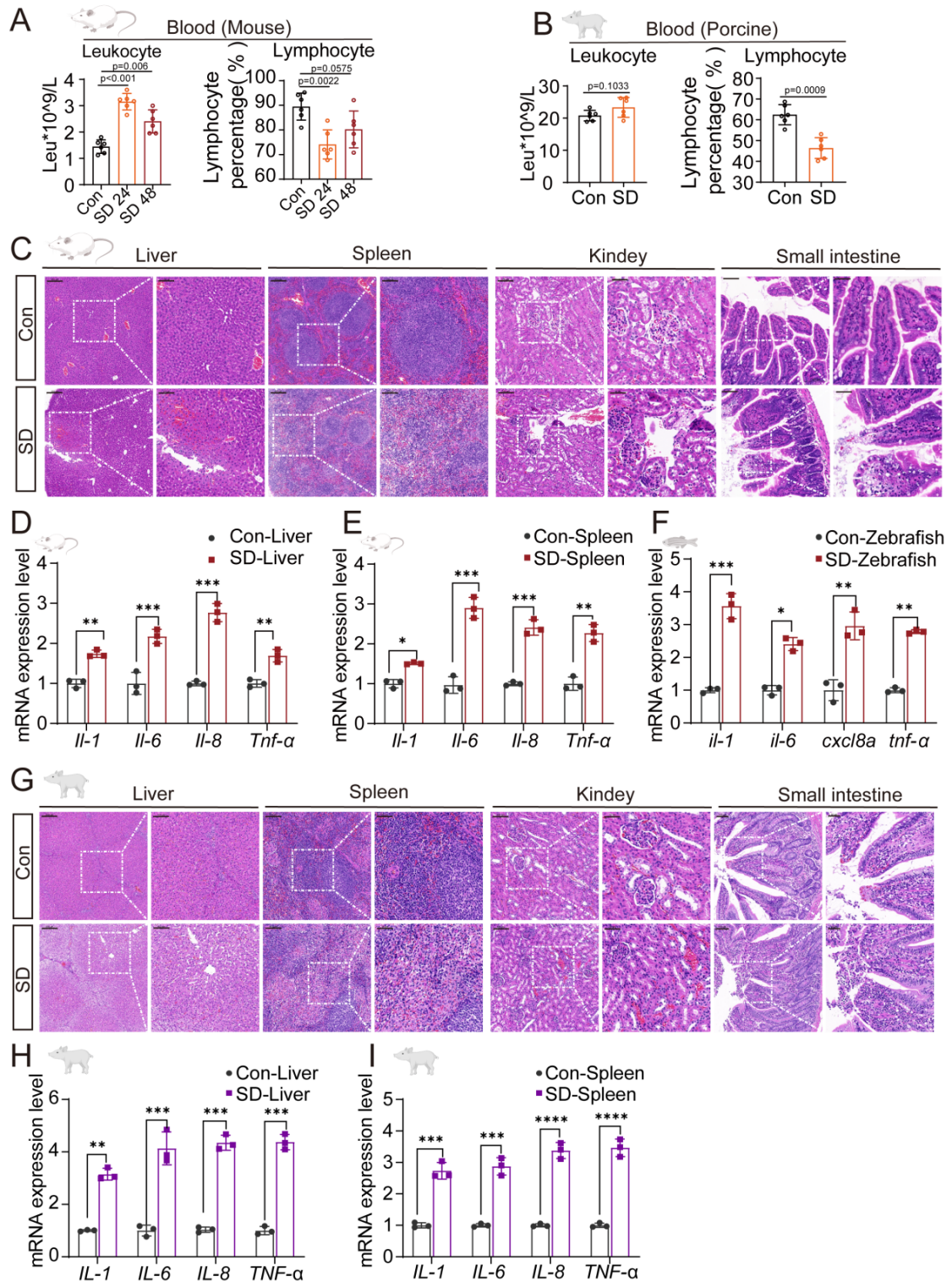
**

**Supplementary Figure 3. SD-induced local tissue inflammation in three model organisms (mice, zebrafish, and pigs). A-B) Quantification of leukocyte counts and lymphocyte percentages in the blood of (A) mice and (B) pigs under control and SD conditions for 96 h. The data are presented as the means ± SD (n = 6/group). C) Representative H&E staining images of liver, spleen, kidney, and small intestine tissues from Con and SD 72 h mice, showing increased inflammatory infiltration in SD-treated mice. Scale bar: 100 µm. D-F) mRNA expression levels of proinflammatory cytokines (*Il-1, Il-6, Il-8,* and *Tnf-α*) in the (D) liver and (E) spleen of mice and (F) 5 dpf zebrafish larvae under Con and SD conditions for 24 h (n = 3 per group). G) Representative H&E staining images of liver, spleen, kidney, and small intestine tissues from Con and SD 96 h pigs, illustrating local tissue inflammation induced by SD. Scale bar: 100 µm. H-I) mRNA expression levels of proinflammatory cytokines (*Il-1, Il-6, Il-8,* and *Tnf-α*) in the (H) liver and (I) spleen of Con and SD pigs (n = 3 per group). Statistical significance was analyzed at each time point via unpaired t tests, with significance levels denoted as follows: ns, p > 0.05; *, p < 0.05; **, p < 0.01; ***, p<0.001;****, p<0.0001.**

**
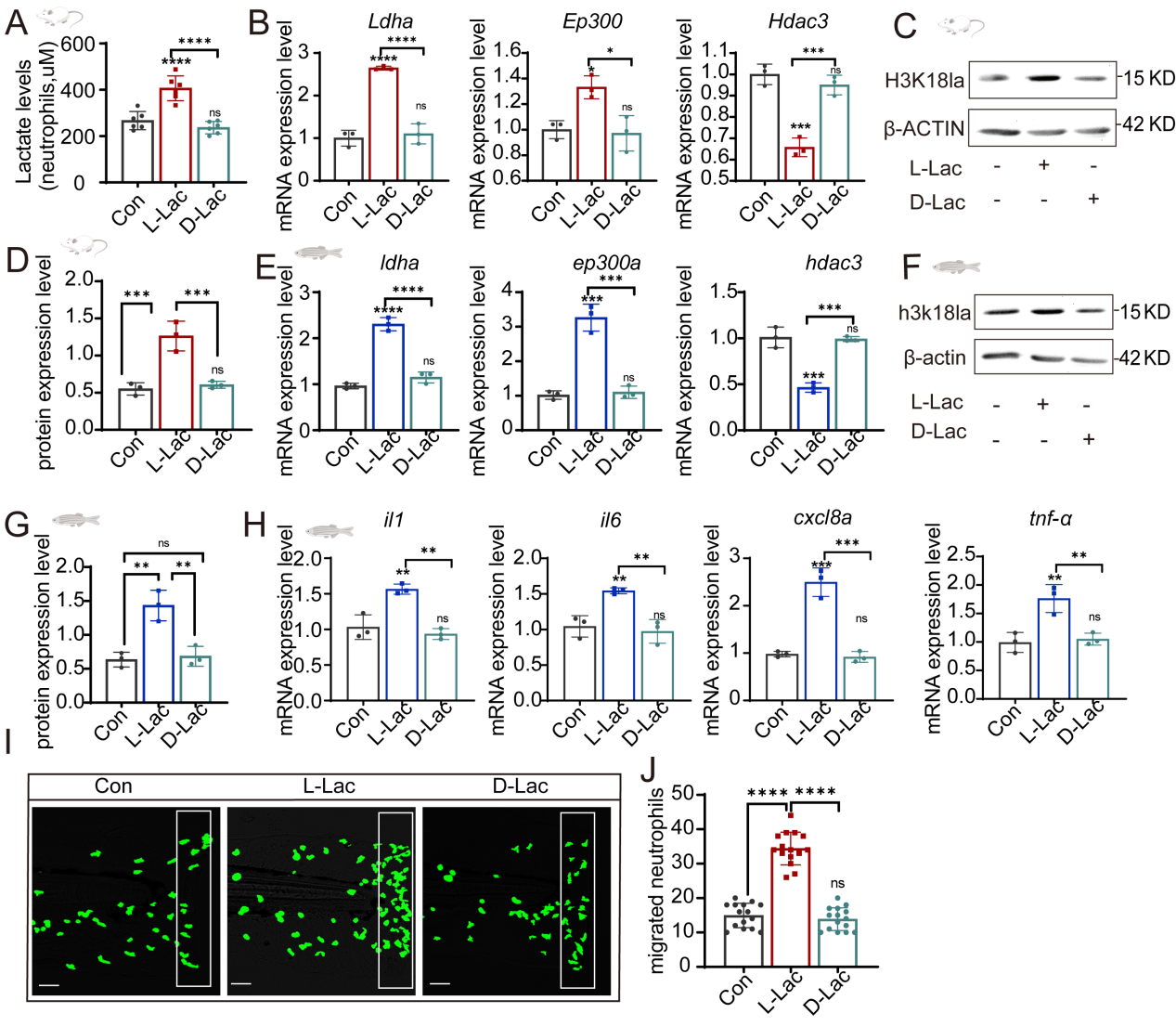
**

**Supplementary Figure 4. Stereospecific effects of L-lactate on lactylation and neutrophil activation in mice and zebrafish. A) Blood neutrophil lactate levels in mice after L-Lac or D-Lac injection (n = 6/group). B) mRNA expression levels of *Ldha, Ep300*, and *Hdac3* in neutrophils from mouse peripheral blood. (n = 3/group). C) Representative western blot showing H3K18la protein levels in neutrophils from mice. D) Quantification of H3K18la protein expression. (n = 3/group). E) mRNA expression levels of *ldha, ep300a,* and *hdac3* in zebrafish larvae following L-Lac or D-Lac treatment. F) Western blot showing H3K18la protein levels in zebrafish. G) Quantification of H3K18la protein levels in zebrafish. H) Expression levels of *il1, il6, cxcl8a,* and *tnf-α* in zebrafish as assessed by RT‒qPCR. I) Representative images of neutrophil migration in the zebrafish caudal fin injury model. Scale bar = 100 μm. J) Quantification of migrated neutrophils per larva. L-Lac, but not D-Lac, significantly increased lactate metabolism, histone lactylation, inflammatory gene expression, and neutrophil migration, confirming the stereospecific effect of L-lactate. The data are shown as the means ± SD. Statistical analyses were performed via one-way ANOVA followed by Tukey’s post hoc test: ns, p > 0.05; *, p < 0.05; **, p < 0.01; ***, p<0.001;****, p<0.0001.**

**
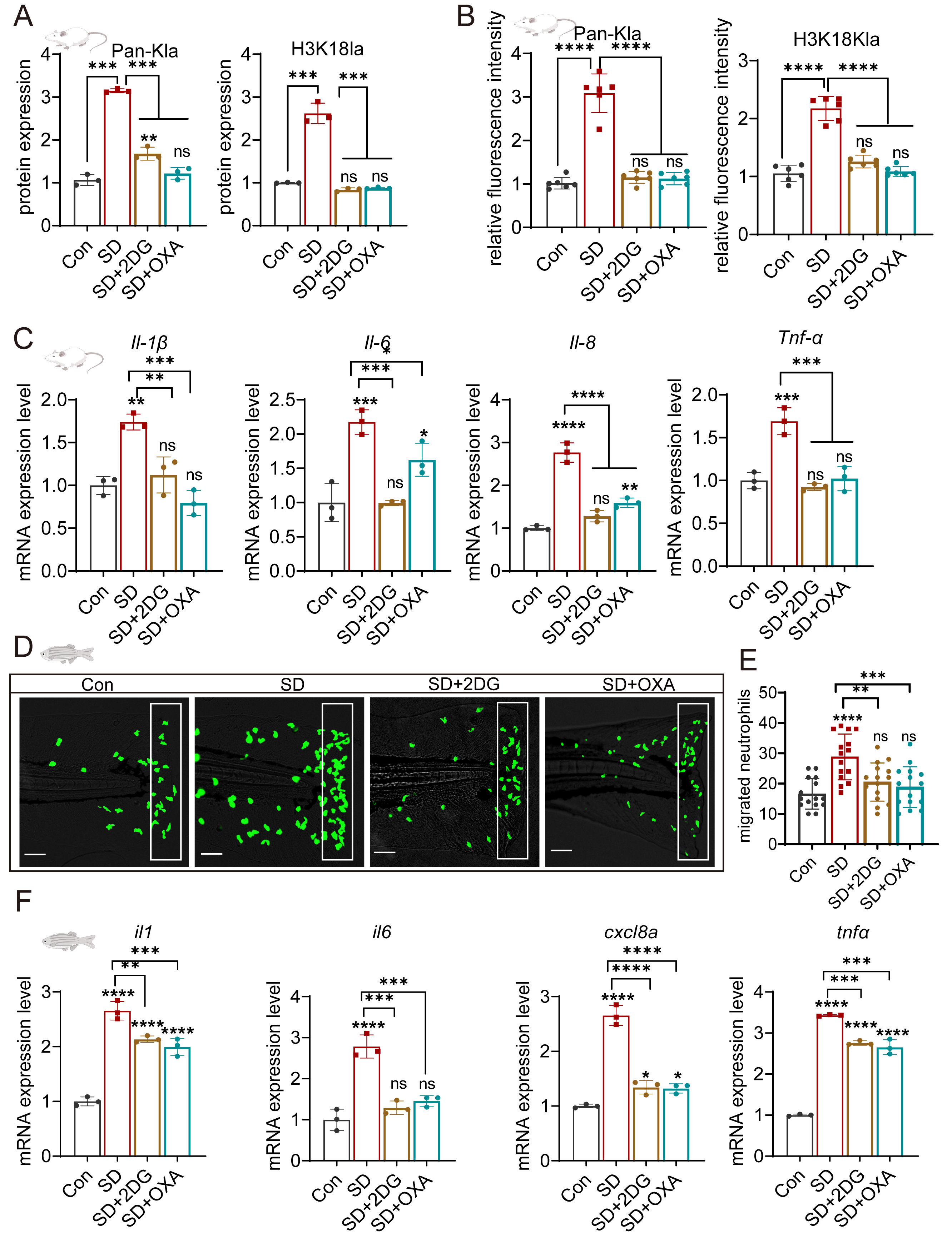
**

**Supplementary Figure 5. Effects of 2DG and OXA intervention on lactylation modifications and neutrophil responses in zebrafish. A) Protein expression of Pan-Kla (lactylation) and H3K18la (histone lactylation) in neutrophils from different treatment groups, as measured by Western blotting (n = 3/group). B) Relative fluorescence intensity of Pan-Kla and H3K18la in neutrophils from zebrafish, as determined by immunofluorescence analysis. The fluorescence intensity indicates the level of lactylation in neutrophils, with significant differences between the treatment groups. C) mRNA expression levels of inflammatory cytokines (*Il-1, Il-6, Il-8,* and *Tnf-α*) in neutrophils isolated from mouse blood following the indicated treatments. Significant changes in gene expression were observed, highlighting the effect of lactylation on inflammatory responses. D) Representative images of neutrophil migration in the caudal fin damage model of zebrafish larvae under different treatment conditions. Neutrophils were stained with a green fluorescence marker, and migration was observed. Scale bars represent 100 µm. E) Quantification of neutrophil migration in zebrafish larvae, showing significant differences between the treatment groups. Migration was significantly greater in the SD and SD + 2DG groups than in the Con and SD + OXA groups. F) mRNA expression levels of inflammatory cytokines (*il-1, il-6, cxcl8a,* and *tnf-α*) in zebrafish larvae following the indicated treatments. Significant differences were observed across the treatment groups, with upregulation in response to lactate-modifying interventions (2DG and OXA). The data in this figure demonstrate the role of lactylation in neutrophil function and the inflammatory response, supporting the influence of 2DG and OXA in regulating immune responses in zebrafish under experimental conditions of sleep deprivation. The data were analyzed via one-way ANOVA with Tukey’s post hoc test, with significance levels denoted as follows: ns, p > 0.05; *, p < 0.05; **, p < 0.01; ***, p<0.001; and ****, p<0.0001.**


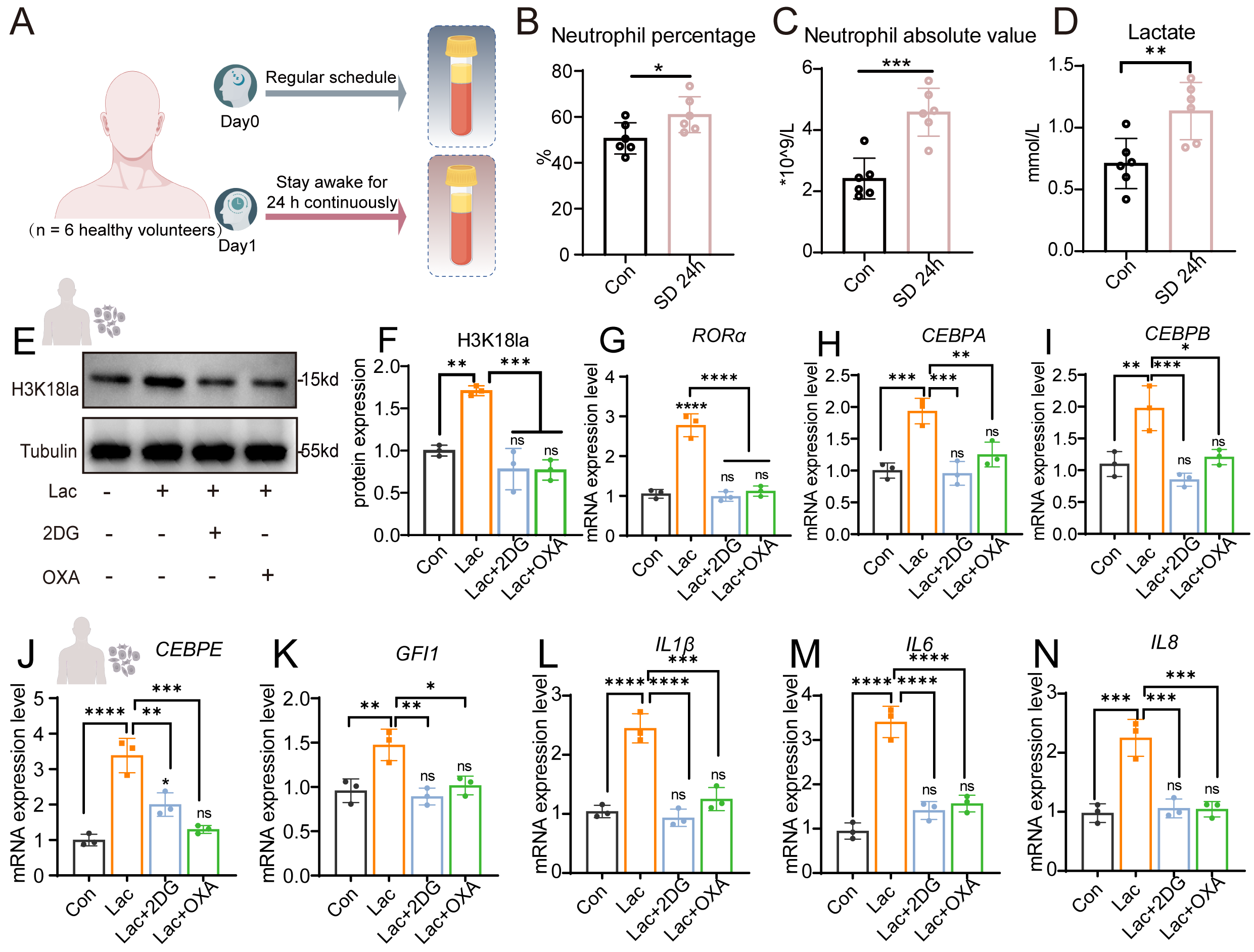


**Supplementary Figure 6. Validation of the lactate–H3K18la–RORα axis in human neutrophil. A)** Schematic of the pilot study in healthy volunteers: peripheral blood was collected after a single night of habitual sleep (Con) or after **24 h of continuous wakefulness** (SD 24 h). **B–D)** Complete blood count data from the same donors (n = 6/group) showing **B)** percentage and **C)** absolute number of circulating neutrophils and **D)** concomitant increase in plasma lactate following acute sleep loss. **E)** Immunoblot analysis of histone H3K18 lactylation (H3K18 la) in commercially sourced primary **human peripheral blood neutrophils** cultured for 4 h under four conditions: vehicle control, sodium L-lactate, lactate + 2-deoxy-D-glucose, or lactate + oxamate. Tubulin, loading control. **F)** Quantification of H3K18la band intensity (n = 3 biological replicates). **G–K)** RT‒qPCR analysis of **RORα** and neutrophil lineage transcription factors (***CEBPA, CEBPB, CEBPE, GFI1***) from the same cultures. **L–N)** Expression of the proinflammatory cytokines ***IL-1β, IL-6,*** and ***IL-8*** under the four metabolic conditions. The data are the means ± SD. One-way ANOVA with Tukey’s post hoc test was used for multicondition comparisons (E–N); paired two-tailed t tests were used for B–D. Significance: ns, p > 0.05; *p < 0.05; **p < 0.01; ***p < 0.001; ****p < 0.0001.
